# Characterization of the phenotype and function of PRELP^+^ fibroblast subtype in liver metastatic colorectal cancer

**DOI:** 10.3389/fgene.2025.1615259

**Published:** 2025-09-15

**Authors:** Yuting Dai, Xingying Huang, Min Sun, Shiyu Zhang, Weiqiang Yu, Kongwang Hu, Qiang Wu, Qingfa Wu

**Affiliations:** ^1^ Center for Advanced Interdisciplinary Science and Biomedicine of IHM, Division of Life Sciences and Medicine, University of Science and Technology of China, Hefei, Anhui, China; ^2^ Department of Pharmacy, The First Affiliated Hospital of USTC, Division of Life Sciences and Medicine, University of Science and Technology of China, Hefei, Anhui, China; ^3^ The First Affiliated Hospital of Anhui Medical University, Hefei, Anhui, China; ^4^ Department of Computer Science, University of Liverpool, Liverpool, United Kingdom; ^5^ HIM-BGI Omics Center, Zhejiang Cancer Hospital, Hangzhou Institute of Medicine (HIM), Chinese Academy of Sciences (CAS), Hangzhou, China

**Keywords:** colorectal cancer, liver metastasis, single-cell RNA-seq, cancer-associated fibroblasts, PRELP^+^ CAF, immunosuppressive TME

## Abstract

**Introduction:**

Fibroblasts are critical mediators of tumor progression and metastasis; however, their heterogeneity and specific functions in the context of colorectal cancer (CRC) liver metastases remain incompletely understood.

**Methods:**

We performed single-cell RNA sequencing (scRNA-seq) analysis on relevant tissue samples. Subsequently, we validated our findings using immunofluorescence assays on histological slides from primary CRC with liver metastasis (mCC) and liver metastatic tumors (mLC). Further investigations included transcriptomic profiling, pseudotime trajectory analysis, multiplex immunofluorescence staining, and cell-cell communication analysis.

**Results:**

Our scRNA-seq analysis identified a distinct PRELP-positive cancer-associated fibroblast (CAF) subtype that is associated with liver metastasis and tumor progression. These PRELP+ CAFs were predominantly enriched in mLC, less abundant in mCC, and rare in non-metastatic CRC (nCC). This distribution was confirmed by immunofluorescence. Transcriptomically, PRELP+ CAFs exhibit a unique signature defined by extracellular matrix components (e.g., PRELP, COLEC11, ITGBL1) and the activation of pro-tumor pathways such as TGF-β and Wnt signaling. Pseudotime analysis indicated they represent a terminal fibroblast differentiation state. Spatially, they colocalize with immune cells (T cells, B cells, plasma cells), and communication analysis suggests they foster an immunosuppressive microenvironment via APP-CD74 and collagen-CD44 signaling, thereby promoting immune evasion. The transcription factors NR2F2, JUN, and JUND were identified as key regulators of this CAF subtype.

**Discussion:**

These findings provide crucial new insights into fibroblast heterogeneity within CRC liver metastases. We characterize PRELP+ CAFs as a specialized, terminally differentiated fibroblast population that contributes to immunosuppression and tumor progression, highlighting them as a potential therapeutic target for inhibiting metastatic advancement.

## 1 Introduction

Fibroblasts are a crucial component of the tumor microenvironment (TME), playing a significant role in cancer progression. Tumor-derived stimuli, including transforming growth factor β (TGF-β), fibroblast growth factor (FGF), platelet-derived growth factor (PDGF), and interleukins, drive the differentiation of normal fibroblasts into cancer-associated fibroblasts (CAFs) (Park et al., 2020). These CAFs exhibit diverse functional states, contributing to tumor growth, metastasis, immune evasion, and extracellular matrix (ECM) remodeling (Chen and Song, 2019; Sahai et al., 2020). One of the primary roles of CAFs in tumor progression is their ability to interact with both tumor cells and stromal cells through cytokines, chemokines, metabolites, and exosomes (Li et al., 2021). For instance, CAFs secrete factors like IL-6, IL-33, and TGF-β, which activate signaling pathways in tumor cells, promoting tumor progression (Qin et al., 2018; Wei et al., 2018; Landskron et al., 2019; Tan et al., 2020; Song et al., 2021). While CAFs are often associated with tumor promotion, recent studies have also highlighted their potential tumor-suppressive roles (Kobayashi et al., 2019; Liu et al., 2019; Menezes et al., 2022). Some CAF subpopulations promote anti-tumor immune responses, activate tumor-suppressive signaling pathways, and restore tumor sensitivity to chemotherapy (Chen et al., 2021). These findings highlight the complex, context-dependent roles of CAFs in cancer progression and treatment responses.

Colorectal cancer (CRC) is the second most common malignant tumor worldwide, with liver metastasis being the most common and lethal form of distant spread (Hackl et al., 2014; Bray et al., 2024). Around 20%–25% of CRC patients present with synchronous liver metastasis, which is associated with poor prognosis and limited treatment options (Manfredi et al., 2006). Advancements in single-cell RNA sequencing (scRNA-seq) have provided valuable insights into the cellular heterogeneity of primary and metastatic CRC (Zhang et al., 2020; Che et al., 2021; Guo et al., 2022; Li et al., 2023). The liver metastatic microenvironment is characterized by an immune-suppressive milieu with reduced immune infiltration, facilitating tumor progression and therapeutic resistance (Liu et al., 2017). scRNA-seq analysis of primary CRC and matched liver metastases revealed a higher abundance of CAFs in primary tumors, whereas a subset of contractile CAFs with a stress-response signature (JUN, BAG3, HSPA2) was exclusive to liver metastases, suggesting adaptation to the metastatic niche (Che et al., 2021). Additionally, liver metastases exhibited dominant MCAM^+^ fibroblasts, which may promote the expansion of CD8 cells expressing the CXCL13 chemokine via Notch signaling, while CD8_CXCL13 cells were associated with high proliferative activity and improved prognosis (Wang et al., 2023). However, the precise functional roles of CAF subtypes in CRC liver metastasis have not been fully understood.

In this study, we performed a comprehensive scRNA-seq analysis and identified the population of PRELP^+^ CAFs significantly enriched in metastatic liver cancers. The proportion of PRELP^+^ CAFs was negatively correlated with patient prognosis. Differential gene analysis revealed that many genes involved in pathways such as TGF-β and Wnt-β signaling, which are associated with proliferation and differentiation, were highly expressed in PRELP^+^ CAFs. Pseudotime trajectory analysis suggested that PRELP^+^ CAFs represent the terminal differentiation state of fibroblasts within liver metastatic tumors. Using multiplex immunofluorescence staining and spatial transcriptomic data, we validated the physical proximity between PRELP^+^ CAFs and immune cells. Additionally, we identified key transcription factors that regulate tumor progression in PRELP^+^ fibroblasts. These findings provide new insights into the role of fibroblasts in tumor progression and metastasis in colorectal cancer.

## 2 Materials and methods

### 2.1 Data sources

Six publicly available single-cell transcriptomics datasets of colorectal cancer were obtained from Gene Expression Omnibus (GEO, https://www.ncbi.nlm.nih.gov/geo/), including GSE205506 (Li et al., 2023), GSE178318 (Che et al., 2021), GSE188711 (Guo et al., 2022), GSE225857 (Wang et al., 2023), GSE144735 (Lee et al., 2020), GSE158692 (Giguelay et al., 2022). To minimize batch effects introduced by different sequencing platforms, all six datasets were generated using the GPL24676 platform (Illumina NovaSeq 6,000, *Homo sapiens*). In total, the scRNA-seq data comprise 112 samples, including tumor tissue, normal tissue, and peripheral blood mononuclear cells (PBMCs) from 48 patients. The clinical information of all samples is summarized in Supplementary Table S1. In addition, one pancreatic ductal adenocarcinoma (PDAC) dataset (GSE197177) and one hepatocellular carcinoma (HCC) dataset (GSE156337) were downloaded from the GEO database.

### 2.2 scRNA-seq data quality control and cell-type annotation

A comprehensive analysis and visualization of single-cell transcriptomics data were conducted using the R package Seurat (version 4.4.0) (Hao et al., 2021), with all cells integrated according to sample ID by Harmony (version 1.2.0) (Korsunsky et al., 2019). To enhance data quality, a filtering criterion was applied, excluding cells with <400 or >50,000 unique molecular identifiers (UMIs) or >25% mitochondrial genes. To further eliminate doublet data, the scDblFinder R package (1.16.0) (Germain et al., 2022) was used. Log-normalization was then applied to standardize the single-cell data using the NormalizeData function, and the top 2,000 most variable genes were identified with the FindVariableFeatures function. After the gene expression matrices were transformed to the natural log scale by the ScaleData function, principal components analysis (PCA) was performed to reduce the dimensionality of the data. The RunHarmony function was subsequently employed to mitigate batch effects and integrate the datasets from the two samples. The number of included components (PCs) was determined based on the ElbowPlot function, with a total of thirty PCs retained. Cells were clustered using the FindNeighbors and FindClusters functions, and resolutions from 0.1 to 0.8 were explored for optimal cell clustering, with the final resolution set to 0.2. For data visualization, the Two-dimensional Uniform Manifold Approximation and Projection (UMAP) was applied using the RunUMAP function. The cells were then split into nine clusters based on commonly used cell markers: TNK cells expressing *CD30* and *NKG7* (n = 17,2941), B cells expressing *CD79A* and *MS4A1* (n = 27,560), plasma cells expressing *CD79A* and *JCHAIN* (n = 47,326), myeloid cells expressing *LYZ* (n = 38,387), endothelial cells expressing *PECAM1* (n = 14,386), fibroblasts expressing *COL1A1* (n = 31,904), epithelial cells expressing *EPCAM* and *KRT8* (n = 51,206), proliferating cells expressing *MKI67* (n = 7,739), and mast cells expressing *TPSB2* (n = 3,907).

To further resolve fibroblast-related subpopulations, a second round of UMAP clustering was conducted. The fibroblast cells were clustered in the 0.5 resolution. Subclusters were identified based on differentially expressed genes using the FindAllMarkers function in Seurat, with cell type annotations assigned according to the most highly expressed genes in each cluster. Among fibroblast-related subpopulations, some clusters encompassed other stromal cell types in addition to fibroblasts. Fibroblasts were distinguished using the markers *DCN* and *LUM*, while pericytes, smooth muscle cells, and glial cells were identified by *RGS5*, *RERGL*, *MYH11* and *GPM6B*, respectively. Additionally, proliferating fibroblasts were characterized by the marker *STMN1*. For the remaining fibroblast subpopulations, cell type annotations were assigned based on differentially expressed genes and highly expressed markers (Du et al., 2024; Gao et al., 2024).

### 2.3 Identification and functional annotation of differentially expressed genes

The FindMarkers function was employed to identify differentially expressed genes (DEGs), and the top 10 DEGs ranked by log_2_FC were selected for heatmap visualization. The R package clusterProfiler (v4.10.1) (Yu et al., 2012) was used for functional annotation of DEGs through the enrichGO and enrichKEGG functions, with the top pathways or terms ranked by -log10(p value) visualized as bar plots or dot plots. The hypergeometric p-value was adjusted using the Benjamini–Hochberg correction. The GSVA R package (v1.40.1) (Hänzelmann et al., 2013) was also utilized, incorporating the hallmark gene sets obtained from the Molecular Signatures Database (MSigDB). Statistical significance was determined using the criteria p.val <0.05 and |avg. logFC| > 0.5.

### 2.4 Tissue distribution of fibroblast subclusters

The distribution of fibroblast subsets across various tissues was analyzed using the ratio of observed to expected cell numbers (Ro/e) (Zhang et al., 2018). This metric was calculated to determine the tissue-specific preference of each subset in three tissues groups. Expected cell numbers for each fibroblast subset-tissue combination were derived using the chi-square test. A fibroblast subset was considered to be enriched in a particular tissue if the Ro/e ratio exceeded 1.

### 2.5 Pseudotime analysis

Monocle2 (v2.28.0) (Qiu et al., 2017) was employed for pseudotime analysis to infer the developmental trajectories of cells within specific cell types based on their gene expression profiles. The UMI matrix was used as input, and variable genes that were detected by dispersionTable function were used for a building trace. A pivotal feature of monocle2, the plot_genes_in_pseudotime function, facilitated the visualization of gene expression changes along pseudotime, representing the inferred developmental trajectory of individual cells. For branch site differential genes analysis of fibroblasts, the BEAM function was used to detect genes that contributed most significantly when cells branched (q < 1 × 10^−4^) and the plot_genes_branched_heatmap function was employed for heatmap visualization.

### 2.6 Cell cycle scoring

Cell cycle phase assignment was performed using the CellCycleScoring function in Seurat (v4.3.0) with the updated human cell cycle gene sets (cc.genes.updated.2019). For each cell, S phase scores were calculated as the average normalized expression of the corresponding gene sets, subtracted by the average expression of the remaining genes.

### 2.7 Cell-cell communication analysis

Cell-cell communication analysis was conducted using the CellChat (v1.6.0) software to model the intercellular pathway network in colorectal cancer. Standardized single-cell data were used as input, incorporating prior knowledge of interacting ligands, receptors, and auxiliary factors for modeling. The interactions between fibroblast subgroups and other cell populations, such as immune cells and epithelial cells, were focused on, with specific emphasis on the expression patterns of ligand-receptor combinations across these cell types. The significance of interactions was calculated using permutation tests. The CellChatDB.human database was employed by loading the “Secreted Signaling Pathways” to infer the cell-cell communication network. The analysis followed the procedures outlined on the CellChat official website (Jin et al., 2021). Centrality measures for each cell type within the network were calculated using the netAnalysis_computeCentrality function to assess their relative importance in cellular communication, while the netAnalysis_signalingRole_network function was used to identify the roles of cells in signal transduction, such as signal senders, receivers, or mediators, within the network.

### 2.8 Survival analysis

The public gene expression data and detailed clinical information were obtained from USUC Xena (https://xena.ucsc.edu/). Survival analysis was performed by R package survival (v3.5–7) and overall survival from the TCGA COADREAD datasets was used. Hazard ratio was calculated by the Cox proportional hazards model and 95% confidence interval was reported, and the Kaplan–Meier survival curve was modeled by survfit function. The Kaplan–Meier survival curves were plotted by using ggsurvplot function and compared using the two-sided long-rank test. Specifically, to test the correlation of each stromal subset and patients’ survival, we used top10 DEGs as gene set for each subset. GSVA enrichment scores for each gene set were calculated using the R package GSVA (v1.50.5) from Bioconductor. The mean of the enrichment scores was then used as a threshold to group samples into high and low groups for downstream survival analysis.

### 2.9 Transcription factor prediction

The single-cell regulatory network inference and clustering (SCENIC) package (v1.1.2.1) (Aibar et al., 2017) was used to analyze the TF activity of fibroblasts from mCC and mLC samples. The super cells were generated by combining the data from every five single cells in each cluster to reduce computing resource consumption. The mean values of normalized counts from five single cells were calculated as the raw input data for SCENIC. The transcription factor prediction website KnockTF2.0 (https://bio.liclab.net/KnockTFv2/index.php) was also used to predict key transcription factors (TFs) of PRELP^+^ CAF clusters in mLC samples.

### 2.10 Immunofluorescence assays

The formalin-fixed paraffin-embedded (FFPE) tissue sections of colorectal cancer and its liver metastasis sites were collected from the Pathology Department of the First Affiliated Hospital of Anhui Medical University. The PRELP^+^ CAFs, Myo Fibroblasts, T cells, B cells and Plasma were characterized by a multi-marker panel in FFPE tissue sections using dual immunofluorescence. Briefly, the sections were deparaffinized in xylene, followed by rehydration in a graded ethanol series. Antigen retrieval was carried out by heating the slides in citrate buffer (pH 6.0) at 95 °C for 30 min. After cooling, the slides were incubated with blocking buffer (5% BSA in PBS) for 1 h at room temperature to reduce nonspecific binding. Rabbit anti-human PRELP (Abcam, ab229719, 1:100) was incubated overnight at 4 °C with mouse anti-human COL1A1 (CST, #6648, 1:200), mouse anti-human aSMA (Fluidgm, 3141017D, 1:200), mouse anti-human CD3 (Santa Cruz, sc-59010, 1:80), mouse anti-human CD138 (Santa Cruz, sc-12765, 1:100), and mouse anti-human CD20 (Servicebio, GB14030-50, 1:150) individually in separate reactions. The following day, secondary antibodies conjugated to different fluorophores (Abcam, ab150077, ab150115,1:100) were applied and incubated for 1 h at room temperature. After washing, the DAPl Staining Solution (biosharp, BL105A) was used to stain the nuclei in the sections. Images were observed with Olympus microscopy and were analyzed with OlyVIA Version 4.1.

## 3 Results

### 3.1 Identification of the PRELP^+^ CAFs enriched in colorectal cancer liver metastases through scRNA-seq analysis

**FIGURE 1:**
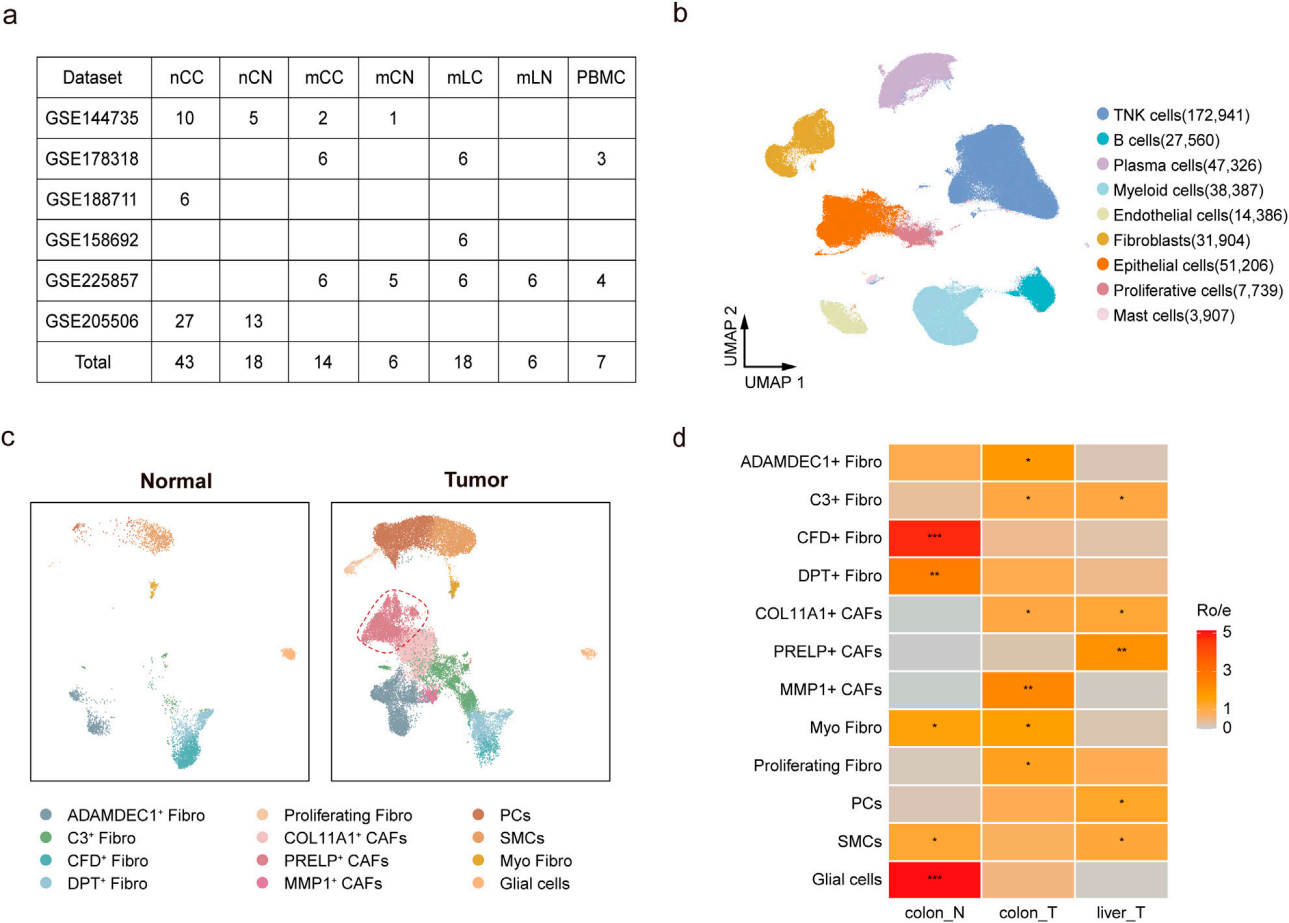
Characterization of cells types in metastatic and non-metastatic colorectal cancer. **(a)** Overview of datasets used in the scRNA-seq analysis. **(b)** UMAP plot showing nine major cell clusters, including TNK cells, epithelial cells, and fibroblasts. **(c)** UMAP plot illustrating 12 fibroblast subpopulations in normal and tumor tissues. **(d)** Relative abundance of fibroblast subsets in colon control (Colon-N, including mCN and nCN), colorectal tumor (Colon-T, including mCC and nCC), and liver metastatic tumor (Liver-T, mLC), estimated using the Ro/e score. Scoring: *(1 < Ro/e ≤ 2), ** (2 < Ro/e ≤ 3), *** (3 < Ro/e ≤ 5).

To investigate fibroblast heterogeneity in primary CRC and liver metastases, we retrieved all relevant single-cell RNA sequencing (scRNA-seq) data from public databases (Lee et al., 2020; Che et al., 2021; Giguelay et al., 2022; Guo et al., 2022; Li et al., 2023; Wang et al., 2023) and conducted a comprehensive scRNA-seq analysis. The dataset comprised 75 tumor samples, 30 adjacent normal tissue samples, and seven peripheral blood mononuclear cell (PBMC) samples from 48 patients (Figure 1a; Supplementary Table S1). We collected clinical information from all samples as much as possible, including age, treatment information, MSI-H/MSS, and AJCC stage information (Supplementary Table S1). Based on histological classification at diagnosis, the samples were categorized as follows: primary CRC without liver metastasis (nCC) and its normal control (nCN), primary CRC with liver metastasis (mCC) and its normal control (mCN), and metastatic liver cancer (mLC) with its normal control (mLN) (Figure 1a; Supplementary Figure S1a).

After quality control and batch effect correction, a total of 440,592 cells were retained for clustering, identifying nine major clusters. The cell types included 75.1% immune cells (47.9% TNK cells, 6.7% B cells, 10.8% plasma cells, and 9.7% myeloid cells), 11.6% epithelial cells, and 7.2% fibroblasts (Figure 1b). These clusters were annotated based on the expression of canonical cell-type marker genes (Supplementary Figure S1c; Supplementary Table S2). Analysis of the cellular composition across samples revealed that PBMC samples were predominantly composed of TNK cells, B cells, and myeloid cells, confirming high-quality cluster classification. Notably, we observed significant heterogeneity in cellular composition between tumor and normal samples, reflecting the inherent variability contributed by individual patient characteristics (Supplementary Figure S1b).

To further investigate the heterogeneity of fibroblasts, we retrieved 31,904 cells from the fibroblast clusters expressing the COL1A1 marker gene and subjected them to additional clustering, resulting in 12 subgroups. Based on marker gene expression, three subgroups were identified as non-fibroblast cells, including glial cells, pericytes (PCs), and smooth muscle cells (SMCs), while the remaining nine subgroups were classified as distinct fibroblast populations (Figure 1c; Supplementary Figure S1d; Supplementary Table S3). We then analyzed the sources of these fibroblast subtypes. MMP1^+^, PRELP^+^, and COL11A1^+^ fibroblasts were derived from tumor tissues and were not found in normal tissues, demonstrating a strong association with tumors and thus being identified as cancer-associated fibroblasts (CAFs). In contrast, ADAMDEC1^+^ Fibro, C3^+^ Fibro, CFD^+^ Fibro, DPT^+^ Fibro, Proliferating Fibro, and Myo Fibro were present in both tumor and normal tissues and were therefore classified under the Fibro group (Figure 1c). Myofibroblasts are typically defined by high expression of *MYH11* and low expression of *RERGL*. In multiple tumor models, fibroblasts exhibiting elevated *MYH11* expression have been reported to play roles in immune regulation, promotion of tumor cell proliferation and migration, as well as inhibition of apoptosis(Grout et al., 2022; Lin et al., 2025). Consistent with previous reports, ADAMDEC1^+^ fibroblasts, characterized by high expression of *ADAMDEC1* and *ADAM28*, are involved in tissue remodeling and healing induced by inflammation (Jasso et al., 2022) and were predominantly found in colorectal samples (Figure 1d). In contrast, a specific fibroblast subgroup, the PRELP^+^ CAFs, was significantly enriched in liver metastasis samples compared to both nCC and mCC samples (Figure 1d; Supplementary Figure S1e,f; Supplementary Table S4). A total of 87 samples in five sample groups contained fibroblasts, and most samples included more than eight cell groups (Supplementary Figure S1f; Supplementary Table S8).

### 3.2 Distinct molecular characteristics and metastasis-promoting role of PRELP^+^ CAFs

**FIGURE 2:**
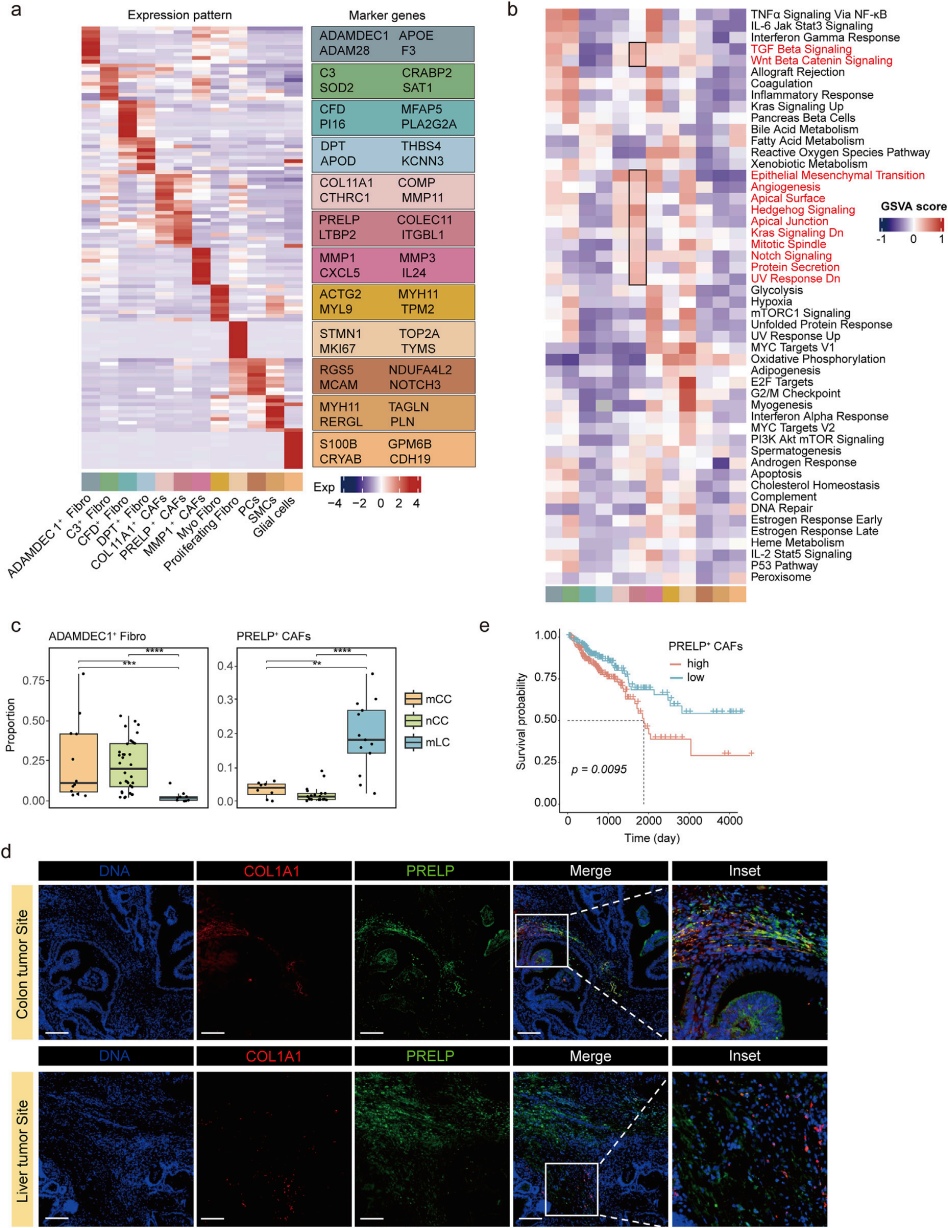
Molecular characteristics and metastasis-promoting role of PRELP^+^ CAFs. **(a)** Heatmap (left) displaying the top 10 upregulated marker genes for each fibroblast subtype, highlighting subtype-specific expression patterns. Fibroblast subtypes are color-coded below the panel. Expression values are represented as z-score normalized means. The right panel lists four representative marker genes. **(b)** Heatmap showing GSVA enrichment scores for 50 hallmark pathways across fibroblast subclusters. Pathways highly enriched in PRELP^+^ CAFs are highlighted in black boxes. Fibroblast subtypes are color-coded as in **(a)**. **(c)** Comparison of ADAMDEC1^+^ Fibro(left) and PRELP^+^ CAF(right) proportions among fibroblasts in nCC, mCC, and mLC. **p < 0.01, ***p < 0.001, Wilcoxon rank-sum test. **(d)** Representative dual immunofluorescence images of PRELP^+^ CAFs in colon and liver metastatic tumors. Magnified views of the boxed regions are shown on the right. Scale bars, 200 μm. **(e)** Kaplan-Meier survival analysis of patients stratified by high (>median) or low (≤median) PRELP^+^ CAF scores.

The top 10 upregulated genes from each fibroblast subtype were considered marker genes, and these marker genes exhibited subtype-specific expression patterns (Figure 2a). We identified four representative marker genes for each fibroblast subtype, consistent with previously characterized fibroblast populations reported in the literature, including ADAMDEC1^+^ fibroblasts (Jasso et al., 2022), CFD^+^ fibroblasts (Du et al., 2024), DPT^+^ fibroblasts (Peng et al., 2022; Peng et al., 2023), COL11A1^+^ CAFs and proliferating fibroblasts (Du et al., 2024). Notably, the expression pattern of the top 10 marker genes in PRELP^+^ CAFs showed some similarity to those in COL11A1^+^ CAFs; however, their overall transcriptional profiles were distinct.

Prolargin (proline/arginine-rich terminal-rich leucine repeat protein), encoded by *PRELP* gene, is a protein that bind type I collagen to basement membranes and type II collagen to cartilage (Lewis, 2003). In most tumors, prolargin is considered a tumor suppressor, but studies in colorectal cancer have shown that it promotes epithelial-mesenchymal transition (EMT) in colorectal cancer cells, which in turn promotes the growth and metastasis of colorectal cancer (Chen et al., 2015; Hong et al., 2020; Dozen et al., 2022; Gui et al., 2024). Other genes highly expressed in PRELP^+^ CAFs also encoded extracellular matrix proteins, including lectins (COLEC11), latent transforming growth factor (TGF)-beta binding proteins (LTBP2), and integrins (ITGBL1) (Figure 2a). GSVA enrichment analysis revealed that many genes in pathways involved in proliferation and differentiation, such as TGF-β signaling and Wnt-β signaling were highly expressed (Figure 2b; Supplementary Figure S2a). Similar to PRELP^+^ CAFs, COL11A1^+^ CAFs were characterized by high expression of extracellular matrix proteins, including COL11A1, COMP, CTHRC1, and MMP11 (Figure 2a; Supplementary Figure S2b). Additionally, COL11A1^+^ CAFs showed increased activity in pathways related to EMT and angiogenesis (Figure 2b). This finding further supports the idea that both PRELP^+^ and COL11A1^+^ CAFs play important roles in the tumor microenvironment, particularly in regulating tumor metastasis and angiogenesis. The pathway enrichment results for PRELP + CAFs in mLC and mCC tissues show that this subpopulation is energetically active in mCC tissues, suggesting that the cells are producing and storing large amounts of energy in preparation for upcoming energy-consuming life activities. In mLC tissues, this subpopulation is enriched in pathways related to extracellular matrix remodeling and adhesion and attachment functions, indicating the functions it performs (Supplementary Figure S3).

Distinct fibroblast distributions were observed across tumor sites. ADAMDEC1^+^ fibroblasts and MMP1^+^ CAFs were more abundant in primary colorectal cancer (mCC and nCC) than in metastatic liver cancer (mLC) (Figure 2c; Supplementary Figure S2c). In contrast, PRELP^+^ CAFs were predominantly found in mLC, with lower proportions in mCC and minimal presence in nCC (Figure 2c). To validate the scRNA-seq analysis results, we performed immunofluorescence assays and examined histological slides of both mCC and mLC samples. We conducted dual immunofluorescence staining using antibodies against PRELP, a protein highly expressed in PRELP^+^ CAFs, and COL1A1, a common fibroblast marker protein. We observed significantly fewer PRELP^+^ CAFs in mCC samples, consistent with scRNA-seq analysis (Figure 2d). This suggests that PRELP^+^ CAFs are associated with advanced colorectal cancer and may contribute to metastasis. Using the top 10 upregulated genes as a signature for PRELP^+^ CAFs, we calculated GSVA score for patients in the TCGA colorectal cancer cohort. Stratification based on these scores revealed that high PRELP^+^ CAF levels were significantly associated with poorer overall survival (Figure 2e), suggesting that PRELP^+^ CAFs play a significant role in tumor progression and advancement.

To further investigate whether CAFs resembling PRELP^+^ CAFs in CRCLM are present in other liver lesions, we analyzed single-cell datasets from pancreatic ductal adenocarcinoma liver metastases (PDADLM) and hepatocellular carcinoma (HCC) (Sharma et al., 2020; Zhang et al., 2023). Since previous studies did not report clusters with features similar to PRELP^+^ CAFs, we re-analyzed fibroblast subpopulations at higher resolution in datasets GSE197177 (PDADLM) and GSE156337 (HCC). In the PDADLM dataset, fibroblasts were clustered into 10 subpopulations. Among these, the C5 cluster showed high expression of PRELP^+^ CAF marker genes—including *COLEC11*, *PRELP*, and *COL10A1* (Supplementary Figure S4a,b,e). However, unlike PRELP^+^ CAFs in CRCLM, which are enriched in liver metastases, the C5 cluster was similarly distributed between primary and metastatic sites (Supplementary Figure S4c). KEGG enrichment analysis further revealed that C5 was primarily associated with immune-related and cellular lifespan pathways—such as apoptosis, TNF signaling, and FoxO signaling—which only partially overlapped with the functional profile of PRELP^+^ CAFs (Supplementary Figure S4g). Thus, we propose that C5 represents a fibroblast subset in PDADLM that shares certain phenotypic features with PRELP^+^ CAFs but differs in tissue distribution and functional emphasis. In contrast, among the five fibroblast subpopulations identified in HCC, none exhibited marker gene expression similar to that of PRELP^+^ CAFs (Supplementary Figure S4d,f). These findings suggest that PRELP^+^ CAFs are not a universal feature of liver-associated fibroblasts, but rather a distinct population specifically associated with colorectal cancer liver metastasis.

### 3.3 PRELP^+^ CAFs represent the terminal differentiation state of fibroblasts in colorectal cancer liver metastases

**FIGURE 3:**
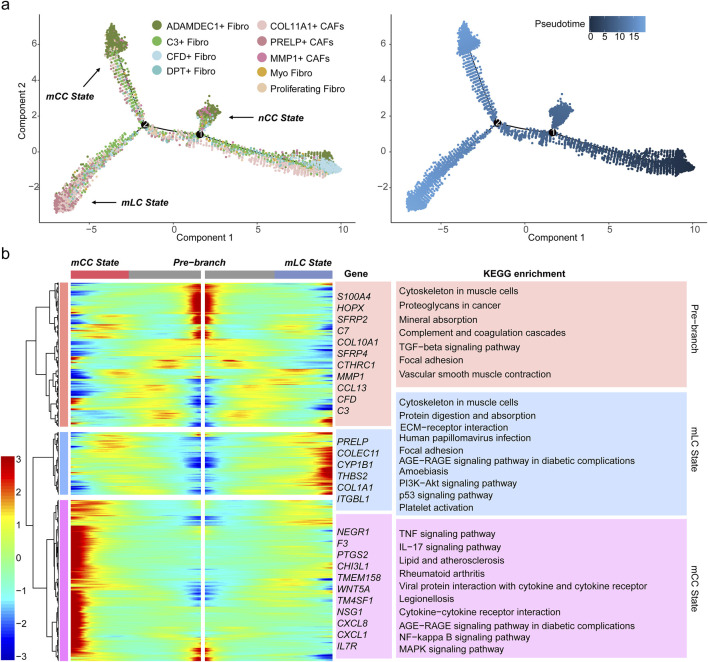
PRELP^+^ CAFs represents a terminal state of fibroblast differentiation. **(a)** Pseudotime trajectory of nine fibroblast subsets, with each dot representing a fibroblast subtype colored accordingly. Three terminal states are indicated (left). The developmental pseudotime progression from progenitor-like fibroblasts (starting at 0) is shown (right). **(b)** Heatmap displaying DEGs across three developmental stages: the initial progenitor stage (pre-branch, center), the mLC state, and the mCC state. Gene expression levels (rows) across fibroblasts (columns) are shown, with cellular states color-coded above. Representative genes are labeled in the middle. The right panel showing the top enriched KEGG pathways associated with DEGs in pre-branch, mLC, and mCC fibroblast states, with background colors corresponding to cellular states.

To investigate the phylogenetic relationships among fibroblast subtypes, we performed pseudotime trajectory analysis using Monocle2, revealing their developmental trajectories. The analysis results for all sample groups indicated a complex differentiation pathway, where fibroblasts transition from initial progenitor-like stages into three distinct terminal states: nCC state, mCC state, and mLC state, as determined by the proportional distribution of fibroblast subtypes across sample groups (Figure 3a; Supplementary Figure S5a-c). The distribution of fibroblast subtypes across these states exhibited distinct preferences. CFD^+^ fibroblasts and DPT^+^ fibroblasts were highly concentrated in the early developmental stages, with their density gradually declining along the trajectory. In contrast, PRELP^+^ CAFs showed a progressive increase in density, reaching their peak at the mLC terminal stage (Figure 3a). Additionally, MMP1^+^ CAFs predominantly occupied the terminal branch of the nCC state, while ADAMDEC1^+^ fibroblasts clustered at the terminal branches of both the nCC state and the mCC state. Meanwhile, COL11A1^+^ CAFs and C3^+^ Fibroblasts were positioned in intermediate transitional states (Figure 3a; Supplementary Figure S5a,b).

Additionally, we examined the expression of proliferative genes across all subpopulations and conducted cell cycle analysis, which further corroborated the pseudotime trajectory results. With the exception of Proliferating Fibro, none of the other subpopulations exhibited high expression of proliferative genes (Supplementary Figure S6a). S-phase scoring revealed that cell clusters at the initial differentiation stage—such as CFD^+^ Fibro and DPT^+^ Fibro—displayed overall higher S-phase scores, suggesting enhanced proliferative and differentiation potential. These populations showed significant differences compared to terminal-stage PRELP^+^ CAFs (Supplementary Figure S6b). The differentiation trends identified in separate analyses of the mCC and mLC sample groups were consistent with those from the integrated analysis. PRELP^+^ CAFs were primarily localized at the terminal end of the differentiation trajectory, although a subset was also observed at intermediate stages (Supplementary Figure S6c). Together, these results indicate a dynamic differentiation process among fibroblast subpopulations, highlighting their distinct functional contributions to colorectal cancer progression and metastasis.

To elucidate the molecular mechanisms underlying these transitions, we identified DEGs along the pseudotime trajectory. These DEGs were clustered into three distinct expression patterns corresponding to the initial developmental stage (pre-branch), mCC state, and mLC state (Figure 3b). The heatmap clearly highlights three distinct gene sets highly expressed in each state, including marker genes for fibroblast subtypes such as PRELP, C3, and CFD, along with numerous functional genes (Figure 3b). To further investigate the biological significance of these genes, KEGG pathway enrichment analysis was performed. The results revealed that genes in the mLC state cluster were primarily enriched in pathways related to ECM organization, ECM-receptor interactions, the PI3K-Akt signaling pathway and the p53 signaling pathway (Figure 3b). The PI3K-Akt signaling pathway is known to promote tumor cell proliferation, survival, and metastasis (Leiphrakpam and Are, 2024). And the p53 signaling pathway mediates the TGFβ–SMAD signaling pathway activation of fibroblasts, which can contribute to an immunosuppressive TME and EMT (Efe et al., 2024). These findings suggest that PRELP^+^ CAFs represent the terminal differentiation state of fibroblasts within liver metastatic tumors, highlighting their role in shaping and maintaining the TME in liver metastases.

### 3.4 PRELP^+^ CAFs contribute to an immunosuppressive tumor microenvironment

**FIGURE 4:**
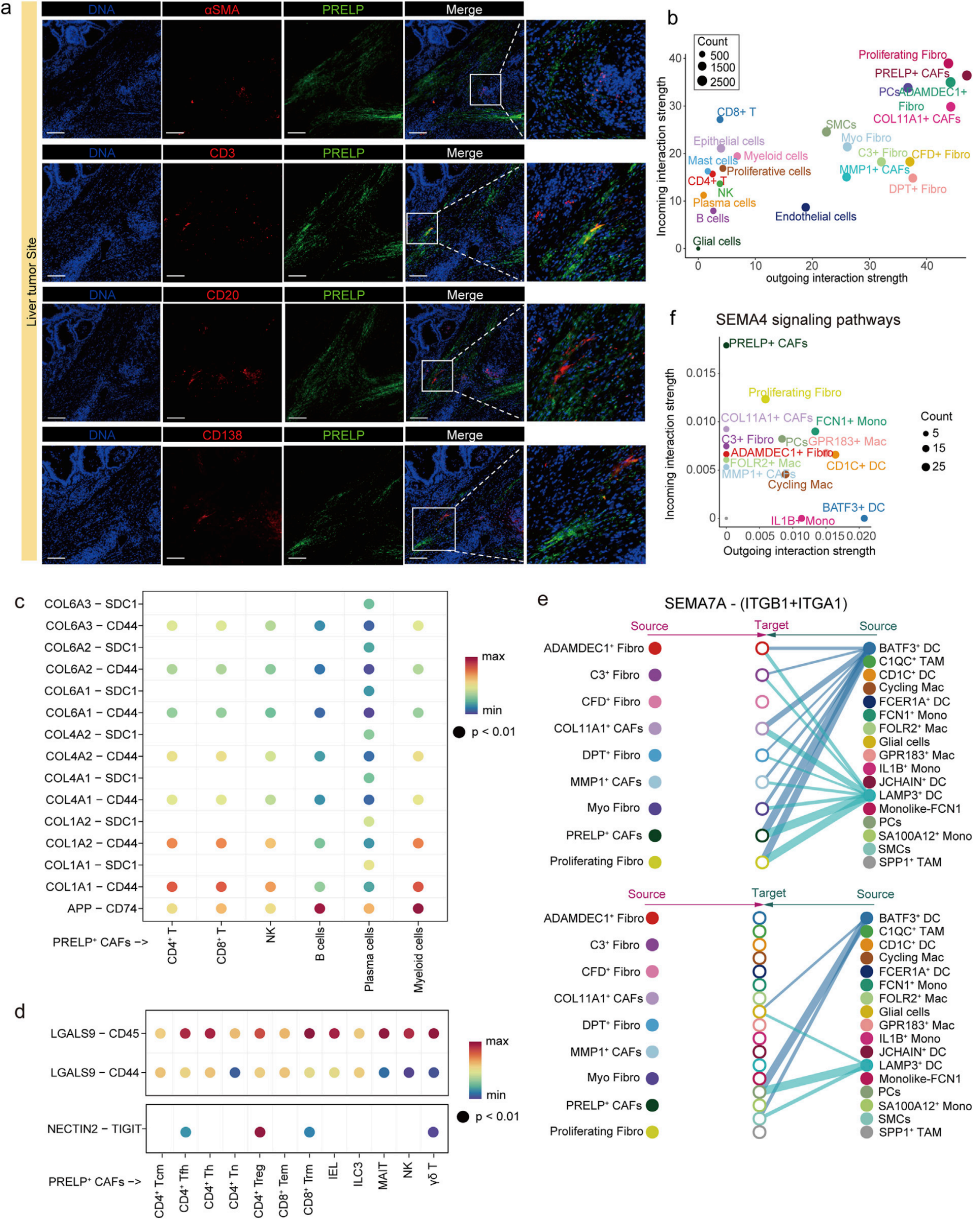
PRELP^+^ CAFs interact and colocalize with immune cells in the tumor microenvironment. **(a)** Immunofluorescence staining in four groups showed spatial colocalization of PRELP (green) with aSMA (red), CD3 (red), CD20 (red) and CD138 (red) in metastatic liver tumors, respectively. Magnified views of the boxed regions are shown on the right. Scale bars, 200 μm. **(b)** Bubble plot illustrating the incoming (vertical axis) and outgoing (horizontal axis) interaction strength of each cell subtype across all signaling pathways. Dot size represents the relative abundance of each cell type. **(c)** Predicted ligand-receptor (L–R) interactions between PRELP^+^ CAFs and six immune cell subtypes in the APP-CD74 axis and the collagen-CD44 axis. Rows indicate specific L-R pairs, and columns represent immune cell subtypes. Dot color denotes the communication probability. **(d)** Predicted ligand-receptor (L–R) interactions of GALECTIN (top) and NECTIN (bottom) signaling pathways between PRELP^+^ CAFs and TNK cell subtypes. Dot color represents the communication probability. **(e)** Hierarchical plots of the SEMA7A/(ITGB1+ITGA1) signaling network inferred by CellChat. Line thickness represents interaction strength and the color of line matches the signal sending cell. In the top panel, fibroblasts act as signal receivers, while in the bottom panel, myeloid cells receive the signals. **(f)** Bubble plot illustrating the incoming (vertical axis) and outgoing (horizontal axis) interaction strength of fibroblast and myeloid cell subtype in SEMA4 signaling pathways. Dot size represents the relative abundance of each cell type.

CAFs contribute to tumor progression by interacting with immune cells through cytokines, chemokines, metabolites, and exosomes (Li et al., 2021). To determine whether PRELP^+^ CAFs are more closely adjacent to immune cells than other subtypes, we performed immunofluorescence staining and compared with Myofibroblasts. The results showed that PRELP^+^ CAFs co-localized spatially with multiple immune cell types (including T cells, B cells, and plasma cells) and were closer to immune cells than Myofibroblasts (Figure 4a). This finding suggests potential interactions between PRELP^+^ CAFs and immune cells within the TME. To further elucidate how PRELP^+^ CAFs modulate the TME, we conducted a cell-cell communication analysis using CellChat on all identified subsets in mLC. To enhance resolution, TNK cells were subdivided into 12 subtypes, while both myeloid and epithelial cells were further clustered into 14 subtypes each (Supplementary Figure S7a; Supplementary Tables S5–S7). This refined classification allowed for a comprehensive evaluation of intercellular signaling dynamics within the TME.

Cellular communication analysis showed that PRELP^+^ CAFs were a highly signaling-active cell type in mLCs, suggesting that they play an important role through strong interactions with other cell types in the tumor microenvironment (Figure 4b). The amyloid precursor protein (APP) is upregulated in colorectal cancer and is linked to increased tumor cell proliferation, migration, and invasion (Pandey et al., 2016). The immunosuppressive effects of the APP-CD74 axis primarily impact B cells and myeloid cells, as observed in testicular tumors (Chen et al., 2024). Additionally, the collagen-CD44 axis plays a key role in mediating interactions between fibroblasts and various immune cells, facilitating the directional migration of immune cells away from the tumor parenchyma and toward the tumor stroma (Yang et al., 2025). Our findings indicate that the APP and collagen signaling pathways mediated by PRELP^+^ CAFs may inhibit immune cell function, promote immune evasion by tumor cells, remodel the ECM, and influence immune cell infiltration and activity through the APP-CD74 and collagen-CD44 axes (Chen et al., 2024; Cheung et al., 2025) (Figure 4c).

Among the 12 TNK cell subsets, PRELP^+^ CAFs exhibited strong signaling, particularly toward CD8^+^ Tem, CD8^+^ Trm, and CD4^+^ Tregs (Supplementary Figure S7b). For example, PRELP^+^ CAFs acted as the primary signal senders in Galectin signaling pathway, with PRELP^+^ fibroblasts generating LGALS9 (Galectin-9). This ligand binds to the glycosylated structure of the CD45 receptor, expressed on all TNK cell subsets (Peng et al., 2024) (Figure 4d; Supplementary Figure S7c). This interaction impairs anti-tumor immune responses and suppresses CD8^+^ Tem proliferation. The TIGIT-NECTIN2 immune checkpoint axis is another key interaction in liver cancer, promoting the creation of an immunosuppressive microenvironment that supports cancer growth (Ho et al., 2021). TIGIT, an inhibitory receptor highly expressed by CD4^+^ Tregs, is activated by NECTIN-2, expressed on PRELP^+^ CAFs (Figure 4d; Supplementary Figure S7c). This activation enhances the immunosuppressive function of Tregs, promoting the secretion of IL-10 and TGF-β(Riquelme et al., 2018). In colorectal cancer research, it has also been reported that cancer-associated fibroblasts (CAFs) can directly suppress effector T cell function, impair proliferation, and promote exhaustion through the TIGIT–NECTIN2 axis, thereby driving immunosuppression (Agorku et al., 2024; Stary et al., 2024). In addition to acting as signal senders in interactions with TNK cells, PRELP^+^ CAFs also function as signal receivers, engaging in crosstalk with myeloid cells. For instance, LAMP3^+^ DCs express SEMA7A, which may regulate gene expression in PRELP^+^ CAFs through SEMA7A-ITGB1/A1 (integrin β1 and α1) interactions. Notably, ITGA1 has been identified as a pro-malignant biomarker in pancreatic cancer, promoting drug resistance and metastatic potential (Gharibi et al., 2017) (Figure 4e). Furthermore, PRELP^+^ CAFs interact specifically with BATF3^+^ DCs, characterized by high expression of CLEC9A, through multiple signaling pathways, including CD226, SEMA4, THY1 and NECTIN (Figure 4f; Supplementary Figure S7d). These findings suggest that the phenotype and function of PRELP^+^ CAFs are shaped by intercellular communication and that they actively contribute to the establishment of an immunosuppressive tumor microenvironment.

### 3.5 Identification of transcription factors driving molecular identity and functional specialization of PRELP^+^ CAFs

**FIGURE 5:**
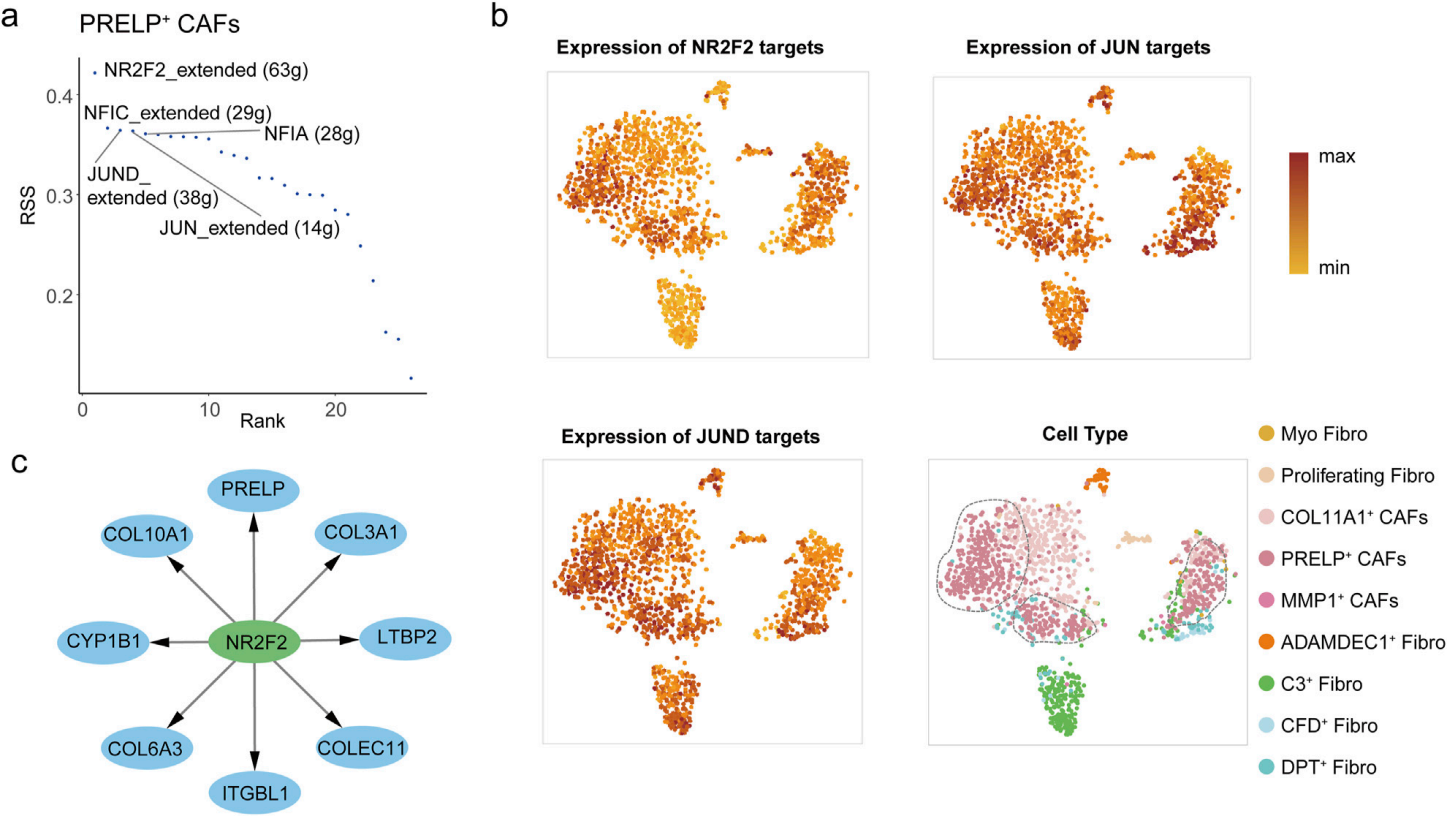
The transcription factors active in PRELP^+^ CAFs. **(a)** Rank score plot showing the top transcription factors regulating gene expression in PRELP^+^ CAFs, ranked by RSS (Regulon Specificity Score). The top5 transcription factors NR2F2, NFIC, JUN, and JUND are highlighted. **(b)** Three UMAP plots illustrate the expression of target genes regulated by NR2F2 (top-left), JUN (top-right), and JUND (bottom-left) in PRELP^+^ CAFs from mLC samples. Dot color represents expression levels, with darker shades indicating higher expression. The remain UMAP plot showing nine fibroblast clusters in mLC samples (bottom-right). Dot color represents the fibroblast cell type. **(c)** Network representation of NR2F2 target genes in PRELP^+^ CAFs, predicted by KnockTF. The central node (NR2F2, green node) is connected to its predicted target genes (blue nodes).

As PRELP^+^ CAFs represent the terminal differentiation state of fibroblasts in colorectal cancer liver metastases, we sought to identify the transcription factors (TFs) driving their distinct phenotype. SCENIC analysis revealed NR2F2, NFIC, JUND, JUN, and NFIA as the top 5 TFs with significantly elevated regulatory activity in PRELP^+^ CAFs compared to other fibroblast subtypes in metastatic liver tumors (Figure 5a; Supplementary Figure S7e). Among these, NR2F2 emerged as the most prominent regulator, with its target genes showing the highest level of activation in PRELP^+^ CAFs (Figure 5b). NR2F2, a ligand-inducible nuclear receptor of the steroid/thyroid hormone receptor superfamily, is mechanistically linked to colorectal cancer progression through its role in TGF-β-dependent EMT, a process critical for tumor cell invasion, metastasis, and poor clinical outcomes (Wang et al., 2015; Wang et al., 2017). To validate the SCENIC findings, we further analyzed the top 50 upregulated DEGs in PRELP^+^ fibroblasts using the KnockTF database to predict potential TF regulators. Remarkably, 90% of these DEGs in PRELP^+^ CAFs were predicted to be regulated by NR2F2, including key markers such as *PRELP*, *COLEC11*, *ITGBL1*, *LTBP2*, and collagen gene (Figure 5C). These ECM-related genes are essential for remodeling the tumor microenvironment and reinforcing the matrix-forming characteristics of PRELP^+^ CAFs. Furthermore, NR2F2 expression strongly correlated with PRELP levels, the defining marker of this CAF subset (Supplementary Figure S7f), solidifying NR2F2 as a key driver of both the molecular identity and functional specialization of PRELP^+^ CAFs.

In addition to NR2F2, the transcription factors JUN (encoding c-JUN) and JUND, members of the AP-1 transcription factor complex, exhibited significant transcriptional activity in PRELP^+^ CAFs (Figure 5b). The AP-1 complex, composed of JUN, FOS, and ATF family proteins, is a well-established mediator of pro-tumorigenic immune modulation (Eferl and Wagner, 2003). Mechanistically, c-JUN shapes the immunosuppressive phenotype of PRELP^+^ CAFs by modulating the expression of key immune-related proteins. For example, c-Jun not only directly binds to the AP-1 regulatory element in the *APP* promoter, activating *APP* expression (Davis, 2015), but has also been reported to enhance *NECTIN2* transcription, a key molecule involved in immune evasion (Lui et al., 2006). Moreover, AP-1 signaling also amplifies TGF-β1 production, which induces galectin-9 expression in colorectal cancer cells (Selnø et al., 2020). Galectin-9 interacts with TIM-3 on immune cells, exacerbating T-cell exhaustion and myeloid cell dysfunction—key mechanisms of immune evasion in metastatic niches.

Together, these findings highlight the intrinsic regulatory mechanisms underlying the diverse functions of PRELP^+^ CAFs, emphasizing their dual roles in stromal remodeling and immunosuppressive regulation within the metastatic tumor microenvironment.

## 4 Discussion

In this study, we conducted a comprehensive scRNA-seq analysis to explore the fibroblast populations within the TME of CRCLM. We identified a specific population of PRELP^+^ CAFs enriched in liver metastatic colorectal cancer (mLC), which were inversely correlated with patient prognosis. This distinct fibroblast population was not as prevalent in primary colorectal cancer with liver metastasis (mCC) or in non-metastatic colorectal cancer (nCC), underscoring their potential role in liver-specific metastasis and tumor progression. Furthermore, we observed a markedly higher abundance of PRELP^+^ CAFs in patients with microsatellite-stable (MSS) tumors—a subtype known for its limited response to immunotherapy—and in those who did not receive neoadjuvant chemotherapy (NAC). This enrichment pattern implies that PRELP^+^ CAFs may contribute to the aggressive phenotype characteristic of MSS colorectal cancers. Based on their functional profile, we hypothesize that PRELP^+^ CAFs promote primary treatment resistance by fostering an immunosuppressive and fibrotic TME that impedes drug delivery and compromises immune cell function. While previous studies have underscored the general protumoral role of fibroblasts, our findings provide novel insights into a specific CAF subpopulation implicated in liver-metastatic CRC, highlighting its potential as a microenvironmental determinant of therapy resistance and disease progression.

CAF subpopulations identified in our study exhibit both conserved and distinct features when compared to previously defined subtypes. ADAMDEC1^+^ fibroblasts, characterized by high expression of ADAMDEC1 and ADAM28, have been implicated in inflammation-driven tissue remodeling and wound healing processes (Jasso et al., 2022). Both CFD^+^ and DPT^+^ fibroblasts display gene signatures typical of tissue-resident fibroblasts with low diversity, which are commonly found in non-malignant adjacent tissues but diminish in abundance within tumor regions (Du et al., 2024). The CFD^+^ subset has also been referred to as PI16^+^ or MFAP5^+^ fibroblasts in earlier studies (Peng et al., 2022; Du et al., 2024). MMP1^+^ CAFs in our dataset showed a pronounced inflammatory phenotype, marked by elevated expression of cytokines, chemokines, and enrichment in inflammatory pathways, consistent with their classification as inflammatory CAFs (iCAFs). The C3^+^ fibroblast subset highly expressed C3, a key component of the complement system, which is known to play a dual role in the tumor microenvironment: it can mediate cytotoxicity against antibody-coated tumor cells and sustain chronic inflammation, yet may also inhibit antitumor T cell responses, thereby potentially supporting tumor progression (Roumenina et al., 2019). This cluster also exhibited elevated expression of CRABP2, which encodes an intracellular lipid-binding protein that influences colorectal cancer proliferation and metastasis through modulation of the MAPK signaling pathway (Yang et al., 2024). Further pathway analysis revealed significant activation of TNF-α/NF-κB and IL-6/JAK/STAT3 signaling, suggesting a potential role for C3^+^ fibroblasts in modulating immune cell activity within the TME.

Consistent with their role in remodeling the TME, PRELP^+^ CAFs exhibited high expression of extracellular matrix components such as *PRELP*, *COLEC11*, *COL10A1*, and *ITGBL1*, as well as genes involved in TGF-β and Wnt signaling pathways, both of which regulate cellular processes crucial for cancer progression like ECM remodeling, cell migration, and immune modulation (Syed, 2016; Zhan et al., 2017). Pseudotime trajectory analysis further suggested that PRELP^+^ CAFs represent a terminally differentiated state of fibroblasts within liver metastatic tumors, underscoring their role in remodeling the TME to facilitate metastatic progression. This observation aligns with the emerging concept that fibroblast differentiation states are dynamically regulated within the TME, and that specific fibroblast subpopulations may adopt distinct functional roles depending on the stage of tumor progression and the tissue context (Kalluri, 2016; Yang et al., 2023).

The terminally differentiated and functionally specialized state of PRELP^+^ CAFs raises the question of their cellular origin within CRCLM. In the liver, CAFs are thought to originate primarily from two physiological sources: resident portal fibroblasts (PFs) and hepatic stellate cells (HSCs) (Baglieri et al., 2019). Previous studies have shown that the origin of CAFs may be related to different tumor pathways to the liver parenchyma, where tumor cells entering the liver through the portal vein may first recruit portal fibroblasts, which then reach a sufficient mass to colonize the liver parenchyma and activate the HSCs, e.g., liver metastases from colorectal cancer, liver metastases from pancreatic cancer. In contrast, hepatocellular carcinoma (HCC) develops in the liver parenchyma, usually in the context of HSC-derived fibrosis, and only contacts the portal interstitial space at an advanced stage, so that the majority of CAFs in HCC originate from HSCs. Furthermore, functional diversity among CAF subtypes may reflect different cellular origins; ECM-rich CAFs are frequently associated with a PF lineage, while contractile CAFs often originate from HSCs or vascular smooth muscle cells (Ramachandran et al., 2019; Giguelay et al., 2022). Notably, in our analysis, a PRELP^+^ CAF-like subpopulation (C5) was detected in PDADLM, but no equivalent population was identified in HCC. Based on these observations, we cautiously speculate that PRELP^+^ CAFs—exhibiting an ECM-producing phenotype—might derive from a portal fibroblast-like lineage rather than an HSC-like origin.

Irrespective of their origin, PRELP^+^ CAFs are strategically positioned to modulate the immune landscape. Our study revealed that PRELP^+^ CAFs are spatially colocalized with various immune cell populations, including T cells, B cells, and plasma cells. This suggests that PRELP^+^ CAFs may play a role in immune modulation within the TME, possibly contributing to the immune-suppressive environment characteristic of liver metastasis. Previous studies have demonstrated that CAFs can influence immune cell behavior through the secretion of cytokines, chemokines, and exosomes, thus promoting immune evasion and tumor progression (Li et al., 2021; Pei et al., 2023). Our findings support this notion, suggesting that PRELP^+^ CAFs may be involved in establishing an immune-tolerant niche that allows tumor cells to evade immune surveillance and promote metastasis. Mechanistically, PRELP^+^ CAFs modulate immune cell function primarily through the APP–CD74 and collagen–CD44 axes. Both interactions have been experimentally validated in prior studies through co-immunoprecipitation, Western blot, affinity chromatography, and blocking assays (Faassen et al., 1992; Ishii et al., 1993; Knutson et al., 1996; Matsuda et al., 2009). Beyond histological evidence in testicular tumors, the APP–CD74 axis was shown to suppress phagocytosis and promote an immunosuppressive phenotype in macrophages in glioblastoma models. Phagocytic function was restored upon APP blockade or knockdown, accompanied by reduced tumor growth (Ma et al., 2024). Meanwhile, the collagen–CD44 axis mediates directional migration of immune cells—diverting cytotoxic lymphocytes away from tumor parenchyma and toward the stroma, thereby facilitating immune escape (Chen et al., 2018; Yuan et al., 2023; Fuller et al., 2024). Elevated CD44 expression is also linked to immunosuppression in gastric cancer, potentially through recruitment of Tregs and M2-like macrophages and upregulation of immune checkpoints (Yang et al., 2025).

The immunosuppressive function of PRELP + CAFs may be driven by members of the JUN family of transcription factors, including JUN and JUND, by regulating the expression of key immune-related molecules. Given their pivotal role in promoting metastasis and immune evasion, targeting PRELP^+^ CAFs or their downstream pathways represents a promising therapeutic strategy for CRC with liver metastasis. Several potential targeting avenues emerge from our findings: the immune checkpoint ligand-receptor pairs APP–CD74 and collagen–CD44, both supported by previous functional evidence and amenable to antibody-mediated or pharmacological inhibition; as well as the upstream regulator NR2F2 (COUP-TFII), for which small-molecule inhibitors such as NR2F2-IN-1 are under investigation to disrupt its transcriptional program. Notably, CD74 is already being clinically targeted with milatuzumab, including in advanced antibody–drug conjugate formats. However, translating these strategies into therapies faces challenges such as tumor heterogeneity, on-target off-tumor effects, and acquired resistance. Thus, while targeting PRELP^+^ CAFs holds considerable promise, further functional studies and rigorously designed clinical trials will be essential to evaluate the efficacy and safety of these approaches.

At the same time, it should be acknowledged that our study is based on retrospective analyses of public datasets and a limited set of tissue samples. Although immunofluorescence staining provided preliminary validation of key results, several limitations remain. First, while the association between PRELP^+^ CAF abundance and poor prognosis is statistically significant, this correlation does not establish causation, and residual confounding from clinical or tumor microenvironmental variables may influence these findings. Second, more in-depth mechanistic investigations—such as functional validation of the CD74/CD44 axis, quantitative assessment of immunosuppressive effects mediated by PRELP^+^ CAFs, and manipulation of candidate transcription factors—were beyond the scope of this study and warrant further investigation in future work.

## Data Availability

Publicly available datasets were analyzed in this study. The datasets can be found in Gene Expression Omnibus (GEO, https://www.ncbi.nlm.nih.gov/geo/), including GSE205506 (Li et al., 2023), GSE178318 (Che et al., 2021), GSE188711 (Guo et al., 2022), GSE225857 (Wang et al., 2023), GSE144735 (Lee et al., 2020), GSE158692 (Giguelay et al., 2022).

## References

[B1] Agorku D. J. Bosio A. Alves F. Ströbel P. Hardt O. (2024). Colorectal cancer-associated fibroblasts inhibit effector T cells via NECTIN2 signaling. Cancer Lett. 595, 216985. 10.1016/j.canlet.2024.216985 38821255

[B2] Aibar S. González-Blas C. B. Moerman T. Huynh-Thu V. A. Imrichova H. Hulselmans G. (2017). SCENIC: single-cell regulatory network inference and clustering. Nat. Methods 14 (11), 1083–1086. 10.1038/nmeth.4463 28991892 PMC5937676

[B3] Baglieri J. Brenner D. A. Kisseleva T. (2019). The role of fibrosis and liver-associated fibroblasts in the pathogenesis of hepatocellular carcinoma. Int. J. Mol. Sci. 20 (7), 1723. 10.3390/ijms20071723 30959975 PMC6479943

[B4] Bray F. Laversanne M. Sung H. Ferlay J. Siegel R. L. Soerjomataram I. (2024). Global cancer statistics 2022: GLOBOCAN estimates of incidence and mortality worldwide for 36 cancers in 185 countries. CA Cancer J. Clin. 74 (3), 229–263. 10.3322/caac.21834 38572751

[B5] Che L. Liu J.-W. Huo J.-P. Luo R. Xu R.-M. He C. (2021). A single-cell atlas of liver metastases of colorectal cancer reveals reprogramming of the tumor microenvironment in response to preoperative chemotherapy. Cell Discov. 7 (1), 80–NA. 10.1038/s41421-021-00312-y 34489408 PMC8421363

[B6] Chen X. Song E. (2019). Turning foes to friends: targeting cancer-associated fibroblasts. Nat. Rev. Drug Discov. 18 (2), 99–115. 10.1038/s41573-018-0004-1 30470818

[B7] Chen R. Dawson D. W. Pan S. Ottenhof N. A. de Wilde R. F. Wolfgang C. L. (2015). Proteins associated with pancreatic cancer survival in patients with resectable pancreatic ductal adenocarcinoma. Lab. Invest 95 (1), 43–55. 10.1038/labinvest.2014.128 25347153 PMC4281293

[B8] Chen C. Zhao S. Karnad A. Freeman J. W. (2018). The biology and role of CD44 in cancer progression: therapeutic implications. J. Hematol. and Oncol. 11 (1), 64. 10.1186/s13045-018-0605-5 29747682 PMC5946470

[B9] Chen Y. McAndrews K. M. Kalluri R. (2021). Clinical and therapeutic relevance of cancer-associated fibroblasts. Nat. Rev. Clin. Oncol. 18 (12), 792–804. 10.1038/s41571-021-00546-5 34489603 PMC8791784

[B10] Chen G. Wang W. Wei X. Chen Y. Peng L. Qu R. (2024). Single-cell transcriptomic analysis reveals that the APP-CD74 axis promotes immunosuppression and progression of testicular tumors. J. Pathol. 264 (3), 250–269. 10.1002/path.6343 39161125

[B11] Cheung B. C. H. Chen X. Davis H. J. Nordmann C. S. Toth J. Hodgson L. (2025). Identification of CD44 as a key engager to hyaluronic acid-rich extracellular matrices for cell traction force generation and tumor invasion in 3D. Matrix Biol. 135, 1–11. 10.1016/j.matbio.2024.11.004 39528207 PMC11729355

[B12] Davis W. Jr. (2015). The ATP-binding Cassette transporter-2 (ABCA2) Overexpression modulates Sphingosine levels and transcription of the amyloid precursor protein (APP) gene. Curr. Alzheimer Res. 12 (9), 847–859. 10.2174/156720501209151019105834 26510981 PMC4630823

[B13] Dozen A. Shozu K. Shinkai N. Ikawa N. Aoyama R. Machino H. (2022). Tumor suppressive role of the PRELP gene in Ovarian clear cell carcinoma. J. Pers. Med. 12 (12), 1999. 10.3390/jpm12121999 36556220 PMC9785654

[B14] Du Y. Shi J. Wang J. Xun Z. Yu Z. Sun H. (2024). Integration of pan-cancer single-cell and spatial transcriptomics reveals stromal cell features and therapeutic targets in tumor microenvironment. Cancer Res. 84 (2), 192–210. 10.1158/0008-5472.CAN-23-1418 38225927

[B15] Efe G. Rustgi A. K. Prives C. (2024). p53 at the crossroads of tumor immunity. Nat. Cancer 5 (7), 983–995. 10.1038/s43018-024-00796-z 39009816

[B16] Eferl R. Wagner E. F. (2003). AP-1: a double-edged sword in tumorigenesis. Nat. Rev. Cancer 3 (11), 859–868. 10.1038/nrc1209 14668816

[B17] Faassen A. E. Schrager J. A. Klein D. J. Oegema T. R. Couchman J. R. McCarthy J. B. (1992). A cell surface chondroitin sulfate proteoglycan, immunologically related to CD44, is involved in type I collagen-mediated melanoma cell motility and invasion. J. Cell Biol. 116 (2), 521–531. 10.1083/jcb.116.2.521 1730766 PMC2289300

[B18] Fuller A. M. Pruitt H. C. Liu Y. Irizarry-Negron V. M. Pan H. Song H. (2024). Oncogene-induced matrix reorganization controls CD8+ T cell function in the soft-tissue sarcoma microenvironment. J. Clin. Invest 134 (11), e167826. 10.1172/JCI167826 38652549 PMC11142734

[B19] Gao Y. Li J. Cheng W. Diao T. Liu H. Bo Y. (2024). Cross-tissue human fibroblast atlas reveals myofibroblast subtypes with distinct roles in immune modulation. Cancer Cell 42 (10), 1764–1783.e10. 10.1016/j.ccell.2024.08.020 39303725

[B20] Germain P. Lun A. Garcia Meixide C. Macnair W. Robinson M. (2022). Doublet identification in single-cell sequencing data using scDblFinder [version 2; peer review: 2 approved]. F1000Research 10 (979), 979. 10.12688/f1000research.73600.2 35814628 PMC9204188

[B21] Gharibi A. La Kim S. Molnar J. Brambilla D. Adamian Y. Hoover M. (2017). ITGA1 is a pre-malignant biomarker that promotes therapy resistance and metastatic potential in pancreatic cancer. Sci. Rep. 7 (1), 10060. 10.1038/s41598-017-09946-z 28855593 PMC5577248

[B22] Giguelay A. Turtoi E. Khelaf L. Tosato G. Dadi I. Chastel T. (2022). The landscape of cancer-associated fibroblasts in colorectal cancer liver metastases. Theranostics 12 (17), 7624–7639. 10.7150/thno.72853 36438498 PMC9691344

[B23] Grout J. A. Sirven P. Leader A. M. Maskey S. Hector E. Puisieux I. (2022). Spatial positioning and matrix programs of cancer-associated fibroblasts promote T-cell exclusion in human Lung tumors. Cancer Discov. 12 (11), 2606–2625. 10.1158/2159-8290.CD-21-1714 36027053 PMC9633420

[B24] Gui Y. Deng X. Li N. Zhao L. (2024). PRELP reduce cell stiffness and adhesion to promote the growth and metastasis of colorectal cancer cells by binding to integrin α5. Exp. Cell Res. 441 (1), 114151. 10.1016/j.yexcr.2024.114151 38992455

[B25] Guo W. Zhang C. Wang X. Dou D. Chen D. Li J. (2022). Resolving the difference between left-sided and right-sided colorectal cancer by single-cell sequencing. JCI Insight 7 (1), e152616. 10.1172/jci.insight.152616 34793335 PMC8765049

[B26] Hackl C. Neumann P. Gerken M. Loss M. Klinkhammer-Schalke M. Schlitt H. J. (2014). Treatment of colorectal liver metastases in Germany: a ten-year population-based analysis of 5772 cases of primary colorectal adenocarcinoma. BMC Cancer 14, 810. 10.1186/1471-2407-14-810 25369977 PMC4230526

[B27] Hänzelmann S. Castelo R. Guinney J. (2013). GSVA: gene set variation analysis for microarray and RNA-seq data. BMC Bioinforma. 14, 7. 10.1186/1471-2105-14-7 23323831 PMC3618321

[B28] Hao Y. Hao S. Andersen-Nissen E. Mauck W. M. 3rd Zheng S. Butler A. (2021). Integrated analysis of multimodal single-cell data. Cell 184 (13), 3573–3587.e29. 10.1016/j.cell.2021.04.048 34062119 PMC8238499

[B29] Ho D. W.-H. Tsui Y.-M. Chan L.-K. Sze K. M.-F. Zhang X. Cheu J. W.-S. (2021). Single-cell RNA sequencing shows the immunosuppressive landscape and tumor heterogeneity of HBV-associated hepatocellular carcinoma. Nat. Commun. 12 (1), 3684. 10.1038/s41467-021-24010-1 34140495 PMC8211687

[B30] Hong R. Gu J. Niu G. Hu Z. Zhang X. Song T. (2020). PRELP has prognostic value and regulates cell proliferation and migration in hepatocellular carcinoma. J. Cancer 11 (21), 6376–6389. 10.7150/jca.46309 33033521 PMC7532499

[B31] Ishii S. Ford R. Thomas P. Nachman A. Steele G. Jr. Jessup J. M. (1993). CD44 participates in the adhesion of human colorectal carcinoma cells to laminin and type IV collagen. Surg. Oncol. 2 (4), 255–264. 10.1016/0960-7404(93)90015-q 7504563

[B32] Jasso G. J. Jaiswal A. Varma M. Laszewski T. Grauel A. Omar A. (2022). Colon stroma mediates an inflammation-driven fibroblastic response controlling matrix remodeling and healing. PLoS Biol. 20 (1), e3001532. 10.1371/journal.pbio.3001532 35085231 PMC8824371

[B33] Jin S. Guerrero-Juarez C. F. Zhang L. Chang I. Ramos R. Kuan C. H. (2021). Inference and analysis of cell-cell communication using CellChat. Nat. Commun. 12 (1), 1088. 10.1038/s41467-021-21246-9 33597522 PMC7889871

[B34] Kalluri R. (2016). The biology and function of fibroblasts in cancer. Nat. Rev. Cancer 16 (9), 582–598. 10.1038/nrc.2016.73 27550820

[B35] Knutson J. R. Iida J. Fields G. B. McCarthy J. B. (1996). CD44/chondroitin sulfate proteoglycan and alpha 2 beta 1 integrin mediate human melanoma cell migration on type IV collagen and invasion of basement membranes. Mol. Biol. Cell 7 (3), 383–396. 10.1091/mbc.7.3.383 8868467 PMC275891

[B36] Kobayashi H. Enomoto A. Woods S. L. Burt A. D. Takahashi M. Worthley D. L. (2019). Cancer-associated fibroblasts in gastrointestinal cancer. Nat. Rev. Gastroenterol. Hepatol. 16 (5), 282–295. 10.1038/s41575-019-0115-0 30778141

[B37] Korsunsky I. Millard N. Fan J. Slowikowski K. Zhang F. Wei K. (2019). Fast, sensitive and accurate integration of single-cell data with Harmony. Nat. Methods 16 (12), 1289–1296. 10.1038/s41592-019-0619-0 31740819 PMC6884693

[B38] Landskron G. De la Fuente Lopez M. Dubois-Camacho K. Diaz-Jimenez D. Orellana-Serradell O. Romero D. (2019). Interleukin 33/ST2 Axis components are associated to Desmoplasia, a metastasis-related factor in colorectal cancer. Front. Immunol. 10, 1394. 10.3389/fimmu.2019.01394 31281317 PMC6598075

[B39] Lee H.-O. Hong Y. Etlioglu H. E. Cho Y. B. Pomella V. Van den Bosch B. (2020). Lineage-dependent gene expression programs influence the immune landscape of colorectal cancer. Nat. Genet. 52 (6), 594–603. 10.1038/s41588-020-0636-z 32451460

[B40] Leiphrakpam P. D. Are C. (2024). PI3K/Akt/mTOR signaling pathway as a target for colorectal cancer treatment. Int. J. Mol. Sci. 25 (6), 3178. 10.3390/ijms25063178 38542151 PMC10970097

[B41] Lewis M. (2003). PRELP, collagen, and a theory of Hutchinson-Gilford progeria. Ageing Res. Rev. 2 (1), 95–105. 10.1016/s1568-1637(02)00044-2 12437997

[B42] Li C. Teixeira A. F. Zhu H.-J. ten Dijke P. (2021). Cancer associated-fibroblast-derived exosomes in cancer progression. Mol. Cancer 20 (1), 154. 10.1186/s12943-021-01463-y 34852849 PMC8638446

[B43] Li J. Wu C. Hu H. Qin G. Wu X. Bai F. (2023). Remodeling of the immune and stromal cell compartment by PD-1 blockade in mismatch repair-deficient colorectal cancer. Cancer Cell 41 (6), 1152–1169.e7. 10.1016/j.ccell.2023.04.011 37172580

[B44] Lin Z. Zhou Y. Liu Z. Nie W. Cao H. Li S. (2025). Deciphering the tumor immune microenvironment: single-cell and spatial transcriptomic insights into cervical cancer fibroblasts. J. Exp. and Clin. Cancer Res. 44 (1), 194. 10.1186/s13046-025-03432-5 40616092 PMC12228347

[B45] Liu Q. Zhang H. Jiang X. Qian C. Liu Z. Luo D. (2017). Factors involved in cancer metastasis: a better understanding to seed and soil hypothesis. Mol. Cancer 16 (1), 176. 10.1186/s12943-017-0742-4 29197379 PMC5712107

[B46] Liu T. Han C. Wang S. Fang P. Ma Z. Xu L. (2019). Cancer-associated fibroblasts: an emerging target of anti-cancer immunotherapy. J. Hematol. Oncol. 12 (1), 86. 10.1186/s13045-019-0770-1 31462327 PMC6714445

[B47] Lui W.-Y. Sze K.-L. Lee W. M. (2006). Nectin-2 expression in testicular cells is controlled via the functional cooperation between transcription factors of the Sp1, CREB, and AP-1 families. J. Cell. Physiology 207 (1), 144–157. 10.1002/jcp.20545 16250013

[B48] Ma C. Chen J. Ji J. Zheng Y. Liu Y. Wang J. (2024). Therapeutic modulation of APP-CD74 axis can activate phagocytosis of TAMs in GBM. Biochim. Biophys. Acta Mol. Basis Dis. 1870 (8), 167449. 10.1016/j.bbadis.2024.167449 39111632

[B49] Manfredi S. Lepage C. Hatem C. Coatmeur O. Faivre J. Bouvier A. M. (2006). Epidemiology and management of liver metastases from colorectal cancer. Ann. Surg. 244 (2), 254–259. 10.1097/01.sla.0000217629.94941.cf 16858188 PMC1602156

[B50] Matsuda S. Matsuda Y. D'Adamio L. (2009). CD74 interacts with APP and suppresses the production of Abeta. Mol. Neurodegener. 4, 41. 10.1186/1750-1326-4-41 19849849 PMC2770512

[B51] Menezes S. Okail M. H. Jalil S. M. A. Kocher H. M. Cameron A. J. M. (2022). Cancer-associated fibroblasts in pancreatic cancer: new subtypes, new markers, new targets. J. Pathol. 257 (4), 526–544. 10.1002/path.5926 35533046 PMC9327514

[B52] Pandey P. Sliker B. Peters H. L. Tuli A. Herskovitz J. Smits K. (2016). Amyloid precursor protein and amyloid precursor-like protein 2 in cancer. Oncotarget 7 (15), 19430–19444. 10.18632/oncotarget.7103 26840089 PMC4991393

[B53] Park D. Sahai E. Rullan A. (2020). SnapShot: cancer-associated fibroblasts. Cell 181 (2), 486–486 e481. 10.1016/j.cell.2020.03.013 32302576

[B54] Pei L. Liu Y. Liu L. Gao S. Gao X. Feng Y. (2023). Roles of cancer-associated fibroblasts (CAFs) in anti- PD-1/PD-L1 immunotherapy for solid cancers. Mol. Cancer 22 (1), 29. 10.1186/s12943-023-01731-z 36759842 PMC9912573

[B55] Peng Z. Ye M. Ding H. Feng Z. Hu K. (2022). Spatial transcriptomics atlas reveals the crosstalk between cancer-associated fibroblasts and tumor microenvironment components in colorectal cancer. J. Transl. Med. 20 (1), 302. 10.1186/s12967-022-03510-8 35794563 PMC9258101

[B56] Peng Z. Ren Z. Tong Z. Zhu Y. Zhu Y. Hu K. (2023). Interactions between MFAP5 + fibroblasts and tumor-infiltrating myeloid cells shape the malignant microenvironment of colorectal cancer. J. Transl. Med. 21 (1), 405. 10.1186/s12967-023-04281-6 37344903 PMC10286363

[B57] Peng H. Jiang L. Yuan J. Wu X. Chen N. Liu D. (2024). Single-cell characterization of differentiation trajectories and drug resistance features in gastric cancer with peritoneal metastasis. Clin. Transl. Med. 14 (10), e70054. 10.1002/ctm2.70054 39422697 PMC11488346

[B58] Qin X. Yan M. Wang X. Xu Q. Wang X. Zhu X. (2018). Cancer-associated fibroblast-derived IL-6 promotes Head and Neck cancer progression via the Osteopontin-NF-kappa B signaling pathway. Theranostics 8 (4), 921–940. 10.7150/thno.22182 29463991 PMC5817102

[B59] Qiu X. Mao Q. Tang Y. Wang L. Chawla R. Pliner H. A. (2017). Reversed graph embedding resolves complex single-cell trajectories. Nat. Methods 14 (10), 979–982. 10.1038/nmeth.4402 28825705 PMC5764547

[B60] Ramachandran P. Dobie R. Wilson-Kanamori J. R. Dora E. F. Henderson B. E. P. Luu N. T. (2019). Resolving the fibrotic niche of human liver cirrhosis at single-cell level. Nature 575 (7783), 512–518. 10.1038/s41586-019-1631-3 31597160 PMC6876711

[B61] Riquelme P. Haarer J. Kammler A. Walter L. Tomiuk S. Ahrens N. (2018). TIGIT+ iTregs elicited by human regulatory macrophages control T cell immunity. Nat. Commun. 9 (1), 2858. 10.1038/s41467-018-05167-8 30030423 PMC6054648

[B62] Roumenina L. T. Daugan M. V. Petitprez F. Sautès-Fridman C. Fridman W. H. (2019). Context-dependent roles of complement in cancer. Nat. Rev. Cancer 19 (12), 698–715. 10.1038/s41568-019-0210-0 31666715

[B63] Sahai E. Astsaturov I. Cukierman E. DeNardo D. G. Egeblad M. Evans R. M. (2020). A framework for advancing our understanding of cancer-associated fibroblasts. Nat. Rev. Cancer 20 (3), 174–186. 10.1038/s41568-019-0238-1 31980749 PMC7046529

[B64] Selnø A. T. H. Schlichtner S. Yasinska I. M. Sakhnevych S. S. Fiedler W. Wellbrock J. (2020). Transforming growth factor beta type 1 (TGF-β) and hypoxia-inducible factor 1 (HIF-1) transcription complex as master regulators of the immunosuppressive protein galectin-9 expression in human cancer and embryonic cells. Aging (Albany NY) 12 (23), 23478–23496. 10.18632/aging.202343 33295886 PMC7762483

[B65] Sharma A. Seow J. J. W. Dutertre C. A. Pai R. Blériot C. Mishra A. (2020). Onco-fetal reprogramming of endothelial cells drives immunosuppressive macrophages in hepatocellular carcinoma. Cell 183 (2), 377–394. 10.1016/j.cell.2020.08.040 32976798

[B66] Song M. He J. Pan Q. Z. Yang J. Zhao J. Zhang Y. J. (2021). Cancer-associated fibroblast-mediated cellular crosstalk supports hepatocellular carcinoma progression. Hepatology 73 (5), 1717–1735. 10.1002/hep.31792 33682185

[B67] Stary V. Pandey R. V. List J. Kleissl L. Deckert F. Kabiljo J. (2024). Dysfunctional tumor-infiltrating Vδ1 + T lymphocytes in microsatellite-stable colorectal cancer. Nat. Commun. 15 (1), 6949. 10.1038/s41467-024-51025-1 39138181 PMC11322529

[B68] Syed V. (2016). TGF-Β signaling in cancer. J. Cell Biochem. 117 (6), 1279–1287. 10.1002/jcb.25496 26774024

[B69] Tan H. X. Gong W. Z. Zhou K. Xiao Z. G. Hou F. T. Huang T. (2020). CXCR4/TGF-β1 mediated hepatic stellate cells differentiation into carcinoma-associated fibroblasts and promoted liver metastasis of colon cancer. Cancer Biol. Ther. 21 (3), 258–268. 10.1080/15384047.2019.1685157 31825725 PMC7012097

[B70] Wang C. Zhou Y. Ruan R. Zheng M. Han W. Liao L. (2015). High expression of COUP-TF II cooperated with negative Smad4 expression predicts poor prognosis in patients with colorectal cancer. Int. J. Clin. Exp. Pathol. 8 (6), 7112–7121. 26261604 PMC4525938

[B71] Wang H. Nie L. Wu L. Liu Q. Guo X. (2017). NR2F2 inhibits Smad7 expression and promotes TGF-β-dependent epithelial-mesenchymal transition of CRC via transactivation of miR-21. Biochem. Biophysical Res. Commun. 485 (1), 181–188. 10.1016/j.bbrc.2017.02.049 28192117

[B72] Wang F. Long J. Li L. Wu Z.-X. Da T.-T. Wang X.-Q. (2023). Single-cell and spatial transcriptome analysis reveals the cellular heterogeneity of liver metastatic colorectal cancer. Sci. Adv. 9 (24), eadf5464. 10.1126/sciadv.adf5464 37327339 PMC10275599

[B73] Wei L. Ye H. Li G. Lu Y. Zhou Q. Zheng S. (2018). Cancer-associated fibroblasts promote progression and gemcitabine resistance via the SDF-1/SATB-1 pathway in pancreatic cancer. Cell Death Dis. 9 (11), 1065. 10.1038/s41419-018-1104-x 30337520 PMC6194073

[B74] Yang D. Liu J. Qian H. Zhuang Q. (2023). Cancer-associated fibroblasts: from basic science to anticancer therapy. Exp. and Mol. Med. 55 (7), 1322–1332. 10.1038/s12276-023-01013-0 37394578 PMC10394065

[B75] Yang R. Yang C. Su D. Song Y. Min J. Qian Z. (2024). METTL3-mediated RanGAP1 promotes colorectal cancer progression through the MAPK pathway by recruiting YTHDF1. Cancer Gene Ther. 31 (4), 562–573. 10.1038/s41417-024-00731-5 38267624 PMC11016466

[B76] Yang Y. Sun H. Yu H. Wang L. Gao C. Mei H. (2025). Tumor-associated-fibrosis and active collagen-CD44 axis characterize a poor-prognosis subtype of gastric cancer and contribute to tumor immunosuppression. J. Transl. Med. 23 (1), 123. 10.1186/s12967-025-06070-9 39871345 PMC11773867

[B77] Yu G. Wang L.-G. Han Y. He Q.-Y. (2012). clusterProfiler: an R Package for comparing biological Themes among gene clusters. OMICS A J. Integr. Biol. 16 (5), 284–287. 10.1089/omi.2011.0118 22455463 PMC3339379

[B78] Yuan Z. Li Y. Zhang S. Wang X. Dou H. Yu X. (2023). Extracellular matrix remodeling in tumor progression and immune escape: from mechanisms to treatments. Mol. Cancer 22 (1), 48. 10.1186/s12943-023-01744-8 36906534 PMC10007858

[B79] Zhan T. Rindtorff N. Boutros M. (2017). Wnt signaling in cancer. Oncogene 36 (11), 1461–1473. 10.1038/onc.2016.304 27617575 PMC5357762

[B80] Zhang L. Yu X. Zheng L. Zhang Y. Li Y. Fang Q. (2018). Lineage tracking reveals dynamic relationships of T cells in colorectal cancer. Nature 564 (7735), 268–272. 10.1038/s41586-018-0694-x 30479382

[B81] Zhang L. Li Z. Skrzypczynska K. M. Fang Q. Zhang W. O’Brien S. A. (2020). Single-cell analyses Inform mechanisms of myeloid-targeted therapies in colon cancer. Cell 181 (2), 442–459. 10.1016/j.cell.2020.03.048 32302573

[B82] Zhang S. Fang W. Zhou S. Zhu D. Chen R. Gao X. (2023). Single cell transcriptomic analyses implicate an immunosuppressive tumor microenvironment in pancreatic cancer liver metastasis. Nat. Commun. 14 (1), 5123. 10.1038/s41467-023-40727-7 37612267 PMC10447466

