# Supplementary material for: Characterization of the phenotype and function of PRELP+ fibroblast subtype in liver metastatic colorectal cancer
Source: Front Genet. 2025 Sep 15;16:1615259. doi: 10.3389/fgene.2025.1615259 (PMC12477005; doi:10.3389/fgene.2025.1615259)
Supplement: Supplementary file 2 [file DataSheet1.docx]

Supplementary Material

# Supplementary Figures


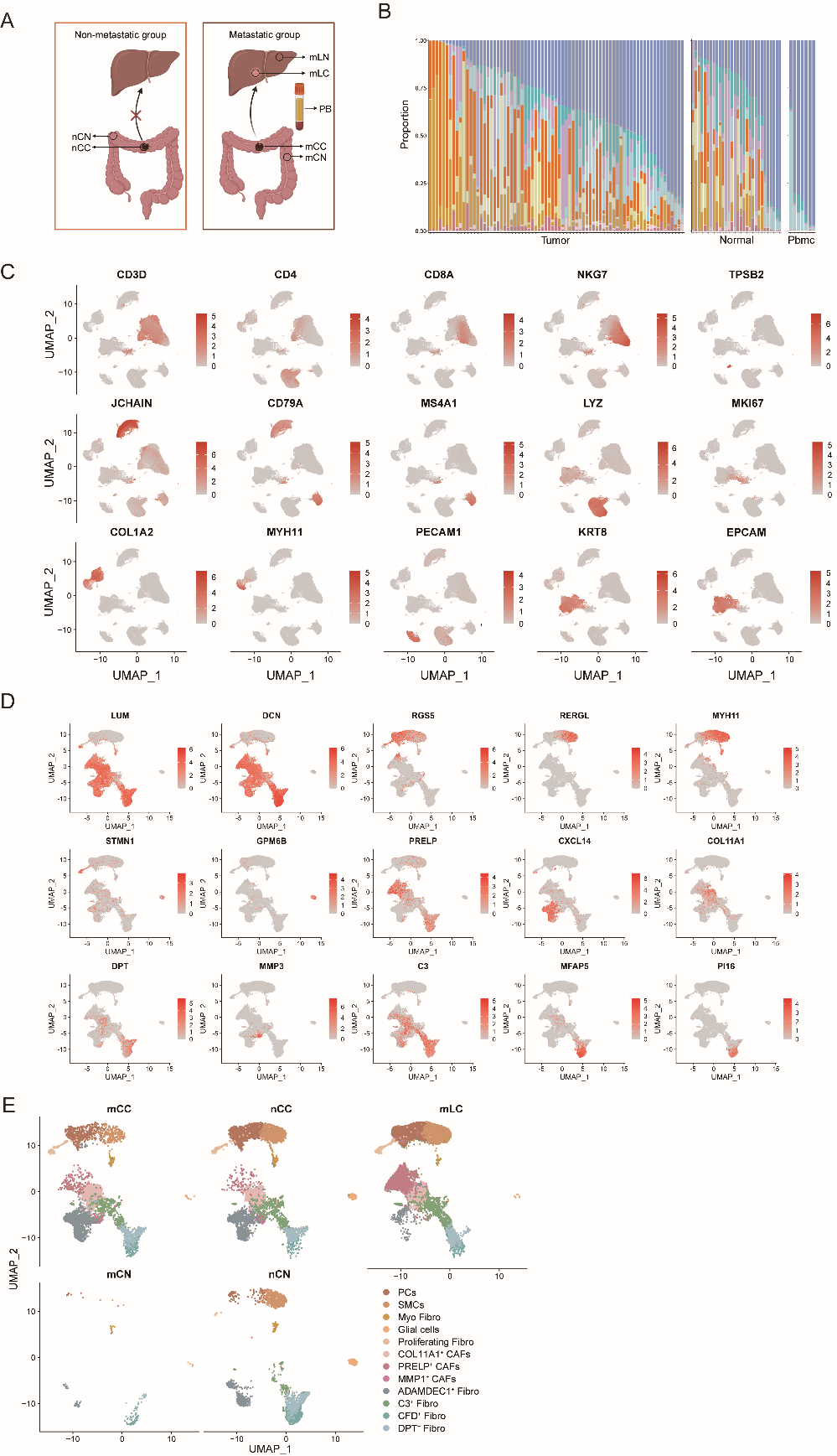


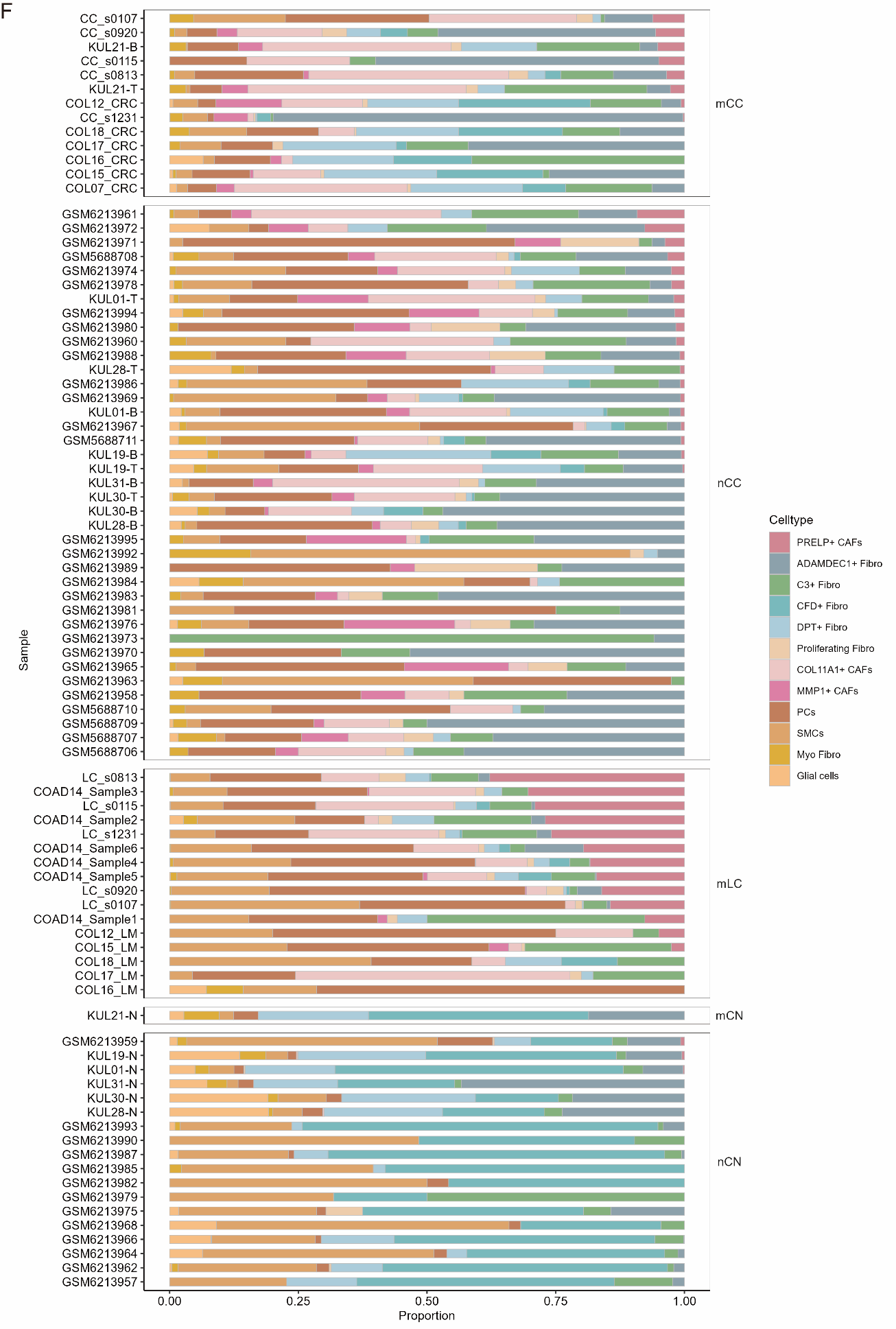


**Supplementary Figure 1.** (A) Schematic representation of sample collection from metastatic and non-metastatic groups. The metastatic group includes metastatic liver normal tissue (mLN), metastatic liver cancer (mLC), peripheral blood (PB), metastatic colorectal cancer (mCC), and metastatic colorectal normal tissue (mCN). The non-metastatic group includes non-metastatic colorectal cancer (nCC) and non-metastatic colorectal normal tissue (nCN). (B) Bar plot showing the proportions of nine major cell clusters across tumor, normal, and PBMC samples. Samples on the x-axis are ordered by increasing TNK cells proportion from right to left. Colors correspond to clusters in Figure1B. (C-D) UMAP plots showing the expression of key marker genes, with (C) highlighting markers for the nine major cell clusters and (D) displaying markers for the 12 fibroblast subtypes. Dot color represents expression levels, with darker red indicating higher expression. (E) UMAP plot illustrating 12 fibroblast subpopulations in five sample groups. (F) Bar plot showing the proportions of 12 fibroblast subpopulations in all samples. Samples on the y-axis are ordered by increasing PRELP⁺ CAFs proportion from top to down.


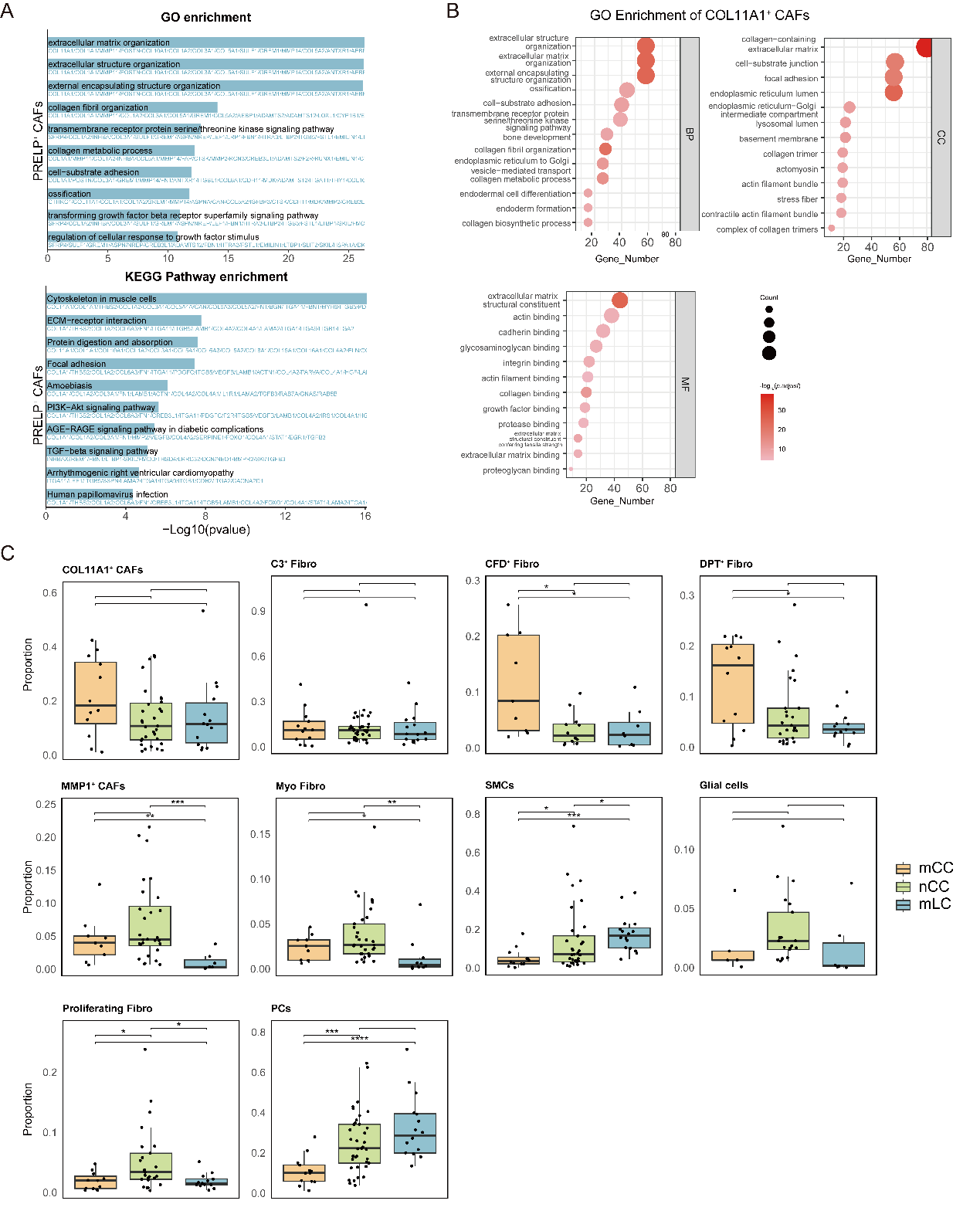


**Supplementary Figure 2.** (A) Bar graphs showing the top 10 pathways identified after GO(top) and KEGG(bottom) enrichment analysis of genes in PRELP^+^ CAFs, ranked by -log10(p-value). (B) Dot plots showing the top GO-enriched terms in COL11A1^+^ CAFs across biological process (BP), molecular function (MF), and cellular component (CC) categories. The size of the dots represents the number of genes, while the color indicates the significance level (-log10(p-value)). (C) Comparison of the proportion of each fibroblast subtype among fibroblasts in nCC, mCC, and mLC. *p < 0.05, **p < 0.01, ***p < 0.001, Wilcoxon rank-sum test.


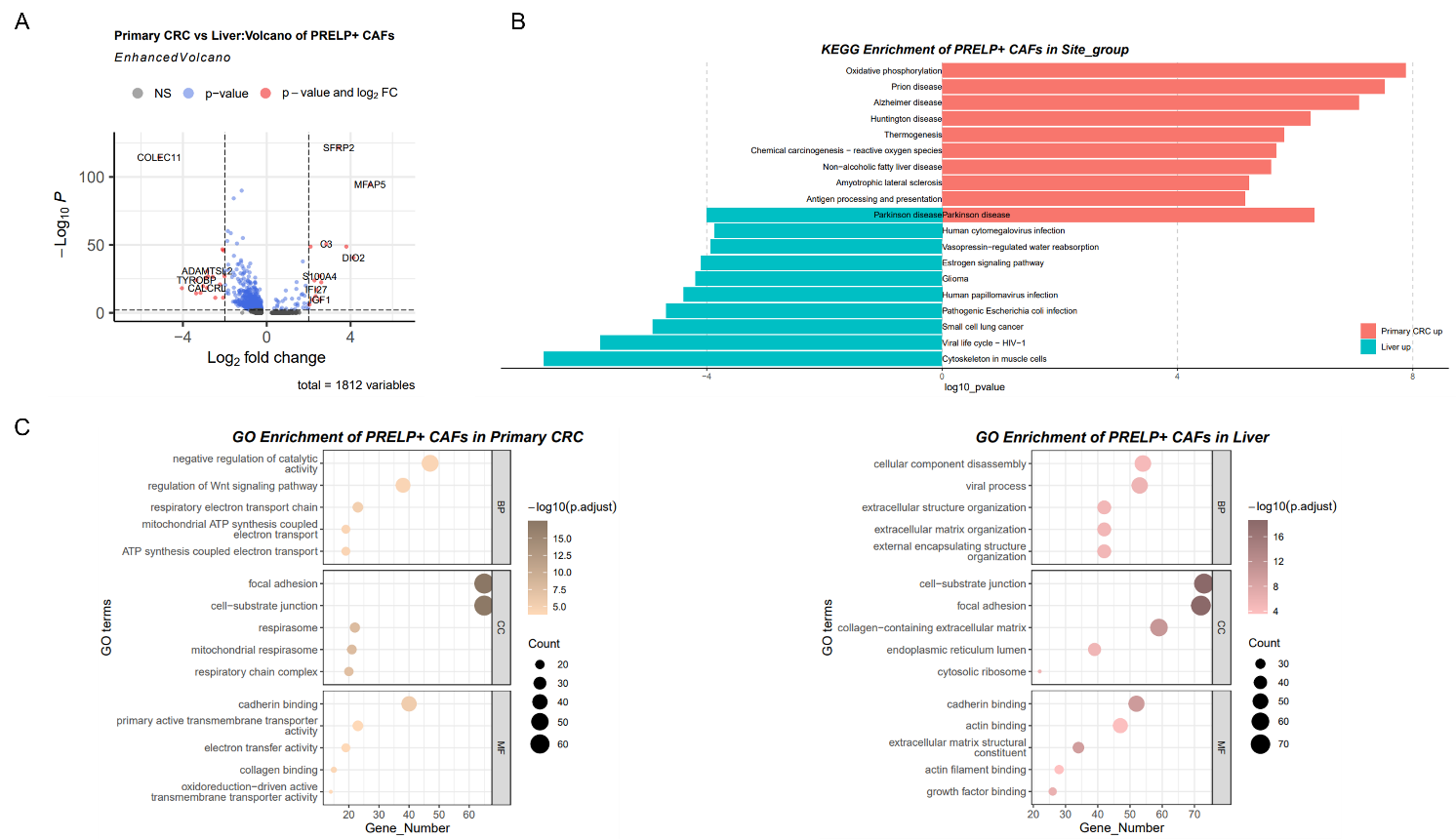


**Supplementary Figure 3.** (A) Volcano plot of differentially expressed genes in the PRELP+ CAFs between mCC (Primary CRC) and mLC (Liver) samples. (B). The bar chart shows the KEGG enrichment results of differentially expressed genes in the PRELP+ CAFs subpopulation between mCC (Primary CRC) and mLC (Liver) samples, displaying only the top ten pathways. The color of the bars represents the sample group, and the length of the bars represents the log10(p-value) value of the pathway enrichment. (C). The bubble chart shows the GO enrichment results of differentially expressed genes in the PRELP+ CAFs subpopulation in mCC (left) and mLC samples (right).

**
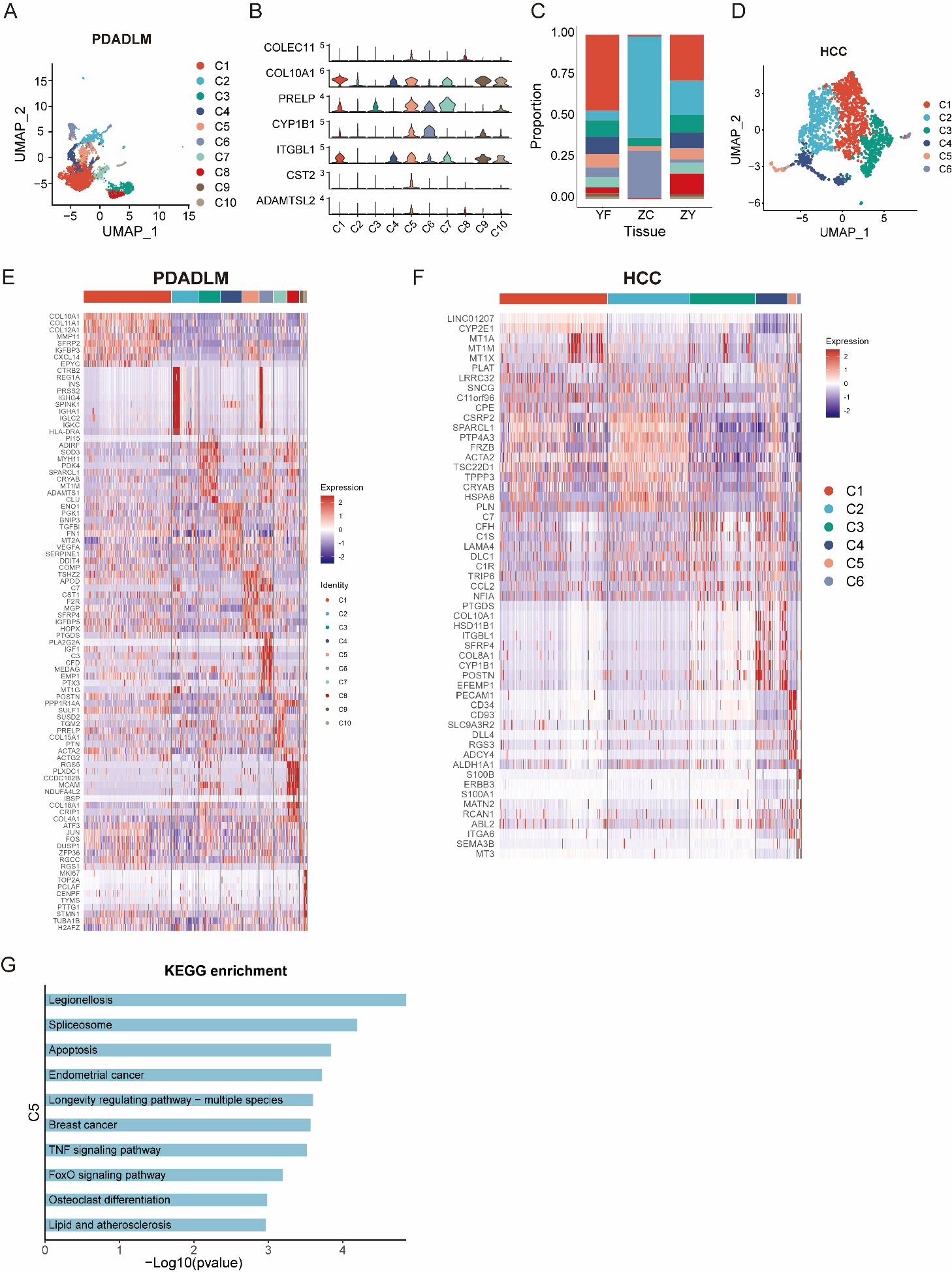
**

**Supplementary Figure 4.** (A) UMAP plots display the 10 fibroblast subsets in the PDADLM. (B) Violin plots displaying the PRELP+ CAFs markers expression pattern across different subtypes of fibroblasts. (C) Bar plot showing the fraction of each fibroblast subset in 3 tissue groups: ZC (normal pancreatic tissue), YF (pancreatic tumors), ZY (hepatic metastases). (D) UMAP plots display the 5 fibroblast subsets in the HCC. (E) Heatmap of top10 DEGs of each fibroblast cluster of PDADLM. (F) Heatmap of top10 DEGs of each fibroblast cluster of HCC. (G) Representative KEGG pathways of the signature genes expressed in C5 clusters. Hypergeometric test was performed with Benjamini–Hochberg adjusted P values.


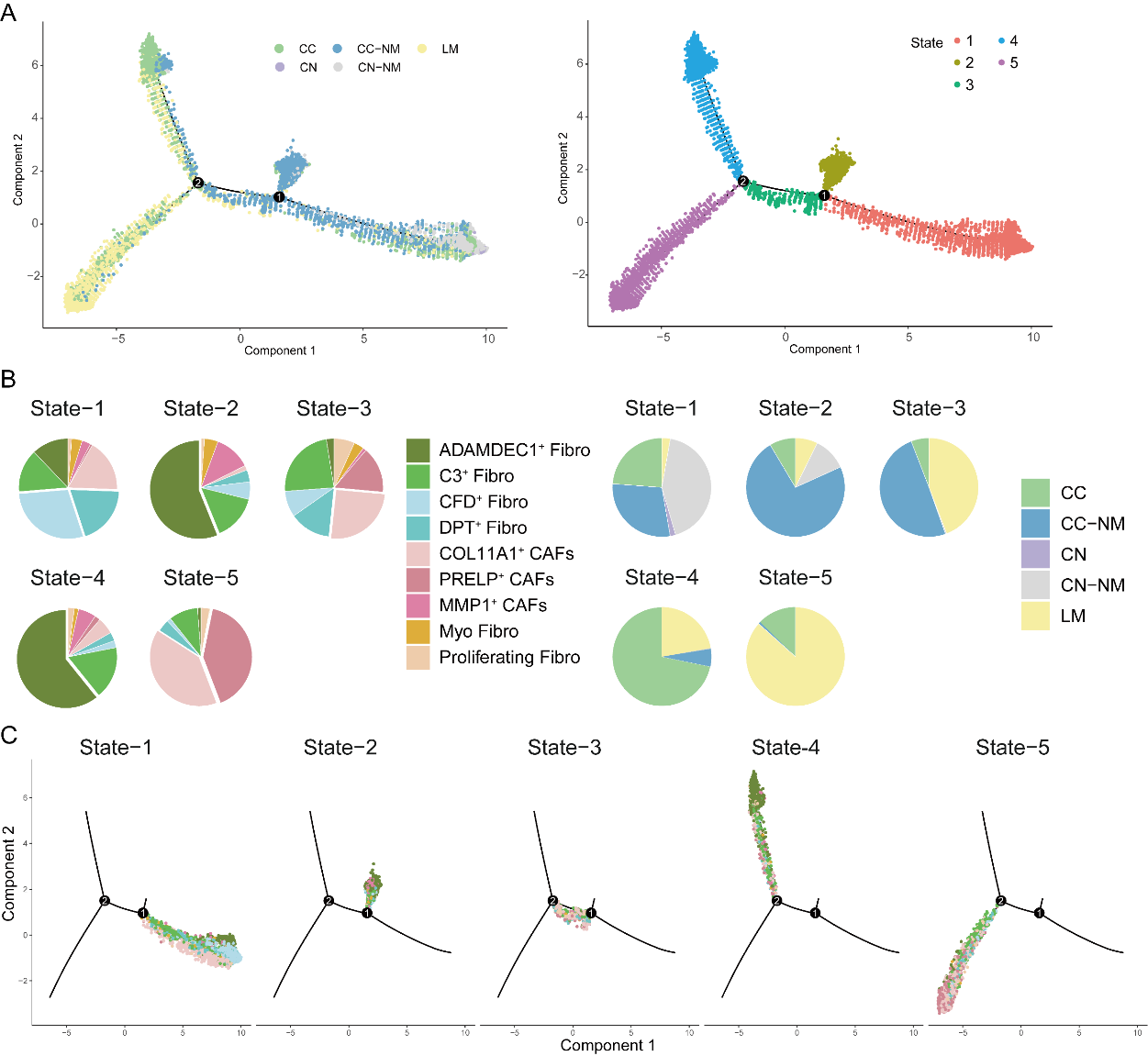


**Supplementary Figure 5.** (A) Semisupervised pseudotime trajectory of nine fibroblast subtypes by Monocle2. Trajectory is colored by cell states (right) and sample groups (left). (B) Pie chart showing the proportion of nine fibroblast subtypes (left) in each cell states. Pie chart showing the proportion of five sample groups (right) in each cell states. (C) Pseudotime trajectories of nine fibroblast subtypes grouped by cell states, repectively. Each panel corresponds to a different state, showing the distribution of fibroblast subtypes within that state.


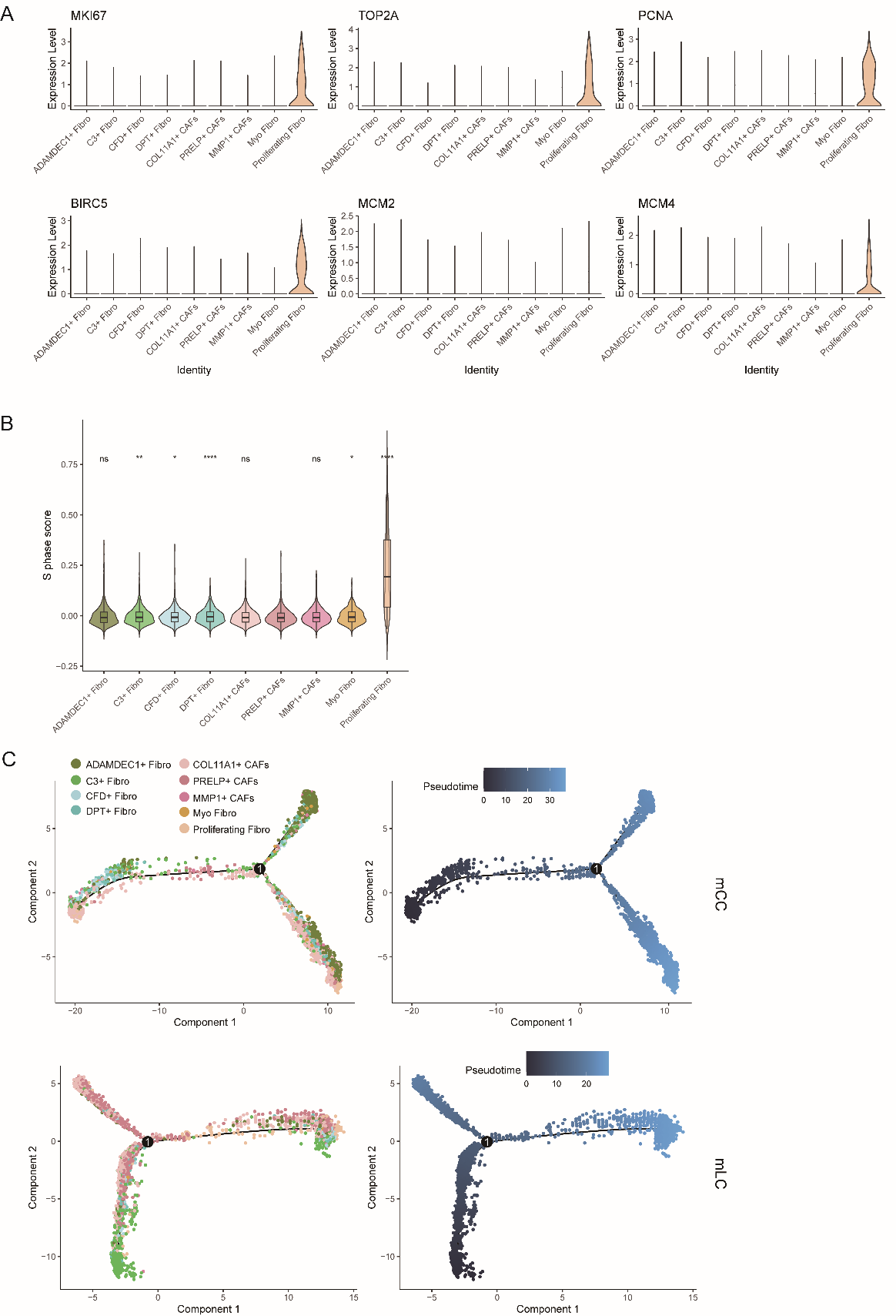


**Supplementary Figure 6.** (A) Violin plots displaying the proliferation genes expression pattern across different subtypes of fibroblasts. (B) Violin plots displaying the S phase score across different subtypes of fibroblasts. ns: p >0.05, *p < 0.05, **p < 0.01, ***p < 0.001, ****p < 0.0001, Wilcoxon rank-sum test. (C) The above are the results of mCC group, and the bottom is the results of mLC group. Pseudotime trajectory of nine fibroblast subsets, with each dot representing a fibroblast subtype colored accordingly(left). The developmental pseudotime progression from progenitor-like fibroblasts (starting at 0) is shown (right).

**
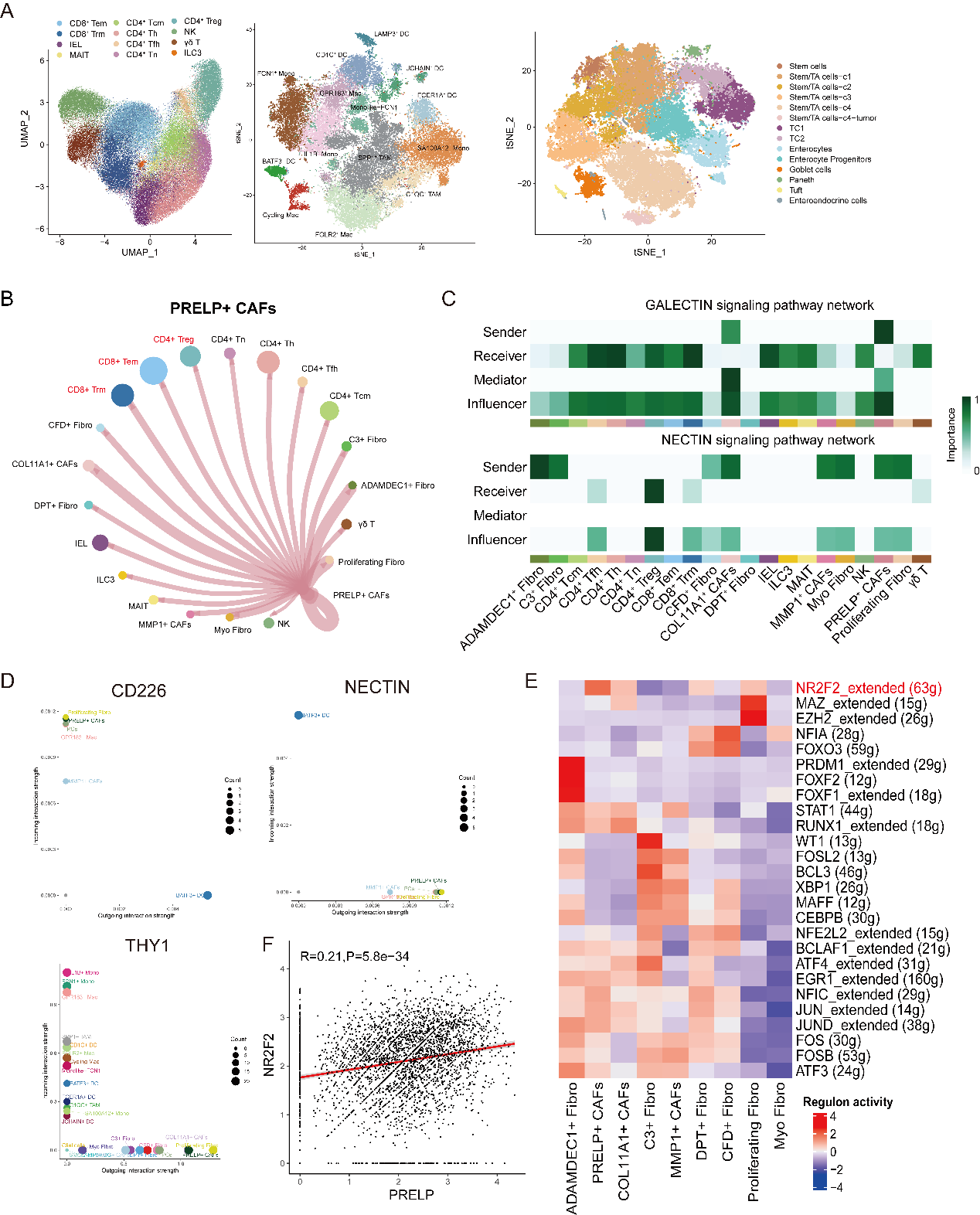
**

**Supplementary Figure 7.** (A) UMAP plot showing 12 TNK subpopulations(left), TSNE plot showing 14 myeloid subpopulations(middle) and 14 epithelial subpopulations(right). (B) Cell-cell communication network centered on PRELP⁺ CAFs, illustrating the interactions with various TNK subpopulations and other fibroblast celltypes. The line thickness represents the interaction strength. (C) Heatmaps showing the importance of different cell types in the GALECTIN and NECTIN signaling pathways. Rows represent sender, receiver, mediator, and influencer roles, while columns indicate different cell types. (D) Bubble plot illustrating the incoming (vertical axis) and outgoing (horizontal axis) interaction strength of fibroblast and myeloid cell subtype in CD226, NECTIN and THY1 signaling pathways. Dot size represents the relative abundance of each cell type. (E) Heatmap of transcription factor regulon activity across fibroblast subtypes, with red-labeled transcription factors NR2F2 representing the highest activity in PRELP⁺ CAFs. (F) Scatter plot showing the correlation between NR2F2 expression and PRELP expression in PRELP+ CAFs from mLC tissue, with a red fitted regression line. The correlation coefficient (R = 0.21) and statistical significance (P = 5.8e-34) are displayed in the upper left corner of the plot.

# Supplementary Table

**Supplementary Table1:** The clinical information of sample; **Supplementary Table2:** The DEGs of 9 celltypes; **Supplementary Table3:** The DEGs of 12 Fibroblast clusters; **Supplementary Table4:** The Ro/e score of fibroblast subclusters; **Supplementary Table5:** The DEGs of 12 TNK clusters; **Supplementary Table6:** The DEGs of 14 myeloid clusters; **Supplementary Table7:** The DEGs of 12 epithelial clusters; **Supplementary Table8:** The Propotion of 12 Fibroblast clusters in each samples.
